# A steric gate controls P/E hybrid-state formation of tRNA on the ribosome

**DOI:** 10.1038/s41467-020-19450-0

**Published:** 2020-11-11

**Authors:** Mariana Levi, Kelsey Walak, Ailun Wang, Udayan Mohanty, Paul C. Whitford

**Affiliations:** 1 Department of Physics, Northeastern University, 360 Huntington Ave, Boston, MA 02115 USA; 2 Department of Chemistry, Boston College, Boston, MA USA; 3 Center for Theoretical Biological Physics, Northeastern University, 360 Huntington Ave, Boston, MA 02115 USA

**Keywords:** Molecular biophysics, Ribosome

## Abstract

The ribosome is a biomolecular machine that undergoes multiple large-scale structural rearrangements during protein elongation. Here, we focus on a conformational rearrangement during translocation, known as P/E hybrid-state formation. Using a model that explicitly represents all non-hydrogen atoms, we simulated more than 120 spontaneous transitions, where the tRNA molecule is displaced between the P and E sites of the large subunit. In addition to predicting a free-energy landscape that is consistent with previous experimental observations, the simulations reveal how a six-residue gate-like region can limit P/E formation, where sub-angstrom structural perturbations lead to an order-of-magnitude change in kinetics. Thus, this precisely defined set of residues represents a novel target that may be used to control functional dynamics in bacterial ribosomes. This theoretical analysis establishes a direct relationship between ribosome structure and large-scale dynamics, and it suggests how next-generation experiments may precisely dissect the energetics of hybrid formation on the ribosome.

## Abstract (web summary)

The ribosome undergoes multiple large-scale structural rearrangements during protein elongation. Here the authors present an all-atom model of the ribosome to study the energetics of P/E hybrid-state formation, an early conformational rearrangement occurring during translocation.

## Introduction

**Fig. 1:**
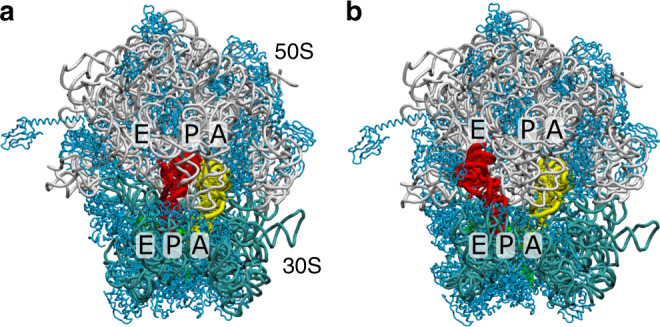
P/E hybrid formation on the ribosome. **a** In the pre-translocation state, tRNA molecules adopt classical A/A and P/P configurations^63^, where one tRNA binds the A sites of the small (30S) and large (50S) subunits, while a second tRNA binds the P sites. The 23S, 16S, mRNA, and proteins are shown in gray, cyan, green, and light blue. **b** In order for the mRNA and tRNA to be displaced between binding sites (i.e. translocation) the P-site tRNA must first adopt a “hybrid” P/E configuration, where it bridges the P site of the small subunit and E site of the large subunit. In addition, the body of the small subunit undergoes a rotation of  ~7°. Structural model of a so-called TI${\,}^{{\rm{Pre}}}$ configuration is shown (see Supplementary Note 1 for details). In the current study, we focus on transitions between P/P and P/E configurations, as well as the impact of A-site tRNA position on P/E formation dynamics.

The bacterial ribosome is a massive (~2.5 MDa) ribonucleoprotein assembly that undergoes large-scale (>50 Å) rearrangements in order to translate messenger RNA (mRNA) sequences into proteins^1–3^. The biological complex is composed of three ribosomal RNA molecules and more than 50 proteins, which arrange to form the large (50S) and small (30S) subunits (Fig. 1). Each cycle of elongation begins with the delivery of an aminoacyl-tRNA (aa-tRNA) molecule to the ribosome by elongation factor Tu. After initial association with the ribosome, the aa-tRNA fully binds the ribosomal A site, a process known as accommodation^4^. This allows a new peptide bond to be formed, after which rotation of the 30S body^5,6^ facilitates the adoption of hybrid conformations^7–9^, where individual tRNAs are displaced between binding sites on the large subunit. Additional rotation^10,11^ and tilting^12^ of the 30S head can then guide the tRNA and mRNA as they move along the 30S subunit (i.e. translocation). Translocation leads to a vacant A site, which enables the ribosome to read the next mRNA codon. With the central role of protein synthesis in the cell, each of these conformational steps must be precisely regulated in order to ensure accurate and efficient expression of the genome.

Experimental studies have provided countless insights into the structure^5,6,10,11,13–18^, timing^8,19–23^ and kinetics^24,25^ of hybrid-state formation and translocation. Translocation involves movement of two tRNA molecules from the A/A–P/P (pre-translocation; Fig. 1a) to P/P–E/E (post-translocation; Supplementary Fig. 1) conformations. The notation N/M refers to a tRNA molecule that is bound to the N (=A, P, or E) site of the small subunit and M site of the large subunit. In order for translocation to occur, the P-site tRNA molecule must first transiently form a hybrid P/E configuration^6,7^, where the anticodon stem loop (ASL) remains bound to the P site of the small subunit, while the acceptor arm and 3′-CCA end associate with the E-site of the large subunit (Fig. 1b). In addition to a  ~20 Å displacement of the tRNA elbow and a  ~40 Å displacement of the 3′-CCA end, reaching the P/E conformation is associated with counterclockwise rotation (6–10°) of the 30S body domain, relative to the 50S subunit^5^. Subsequently^16,26^, or perhaps simultaneously^27^, the A-site tRNA adopts A/P-like hybrid configurations^9,28^, where it contacts the A site of the small subunit and P site of the large subunit. The tRNA molecules then proceed to the P/P–E/E conformation, which completes the translocation process.

Even though computational and experimental approaches have advanced significantly over the last decade, the transition states that control P/E formation kinetics have yet to be precisely identified. This gap represents an opportunity for energy landscape techniques to help reveal the interactions that shape the underlying free-energy barriers, and therefore control the biological kinetics. The power of energy landscape approaches is exemplified by applications to biomolecular folding, where comparison of theory and experiments has provided an understanding of the physical principles that govern dynamics^29–33^. Inspired by successes in folding, landscape approaches have been adopted to study a range of biological phenomena, including assembly^34^, chromatin remodeling^35^ and cell motility^36^, as well as ribosome dynamics^16,37^. With ever-increasing computing capabilities and the development of novel theoretical models, it is also now possible for simulations to partition the energetic contributions of steric/entropic factors^38^, electrostatic interactions^39^, and chemical steps^40,41^ in the ribosome.

**Fig. 2:**
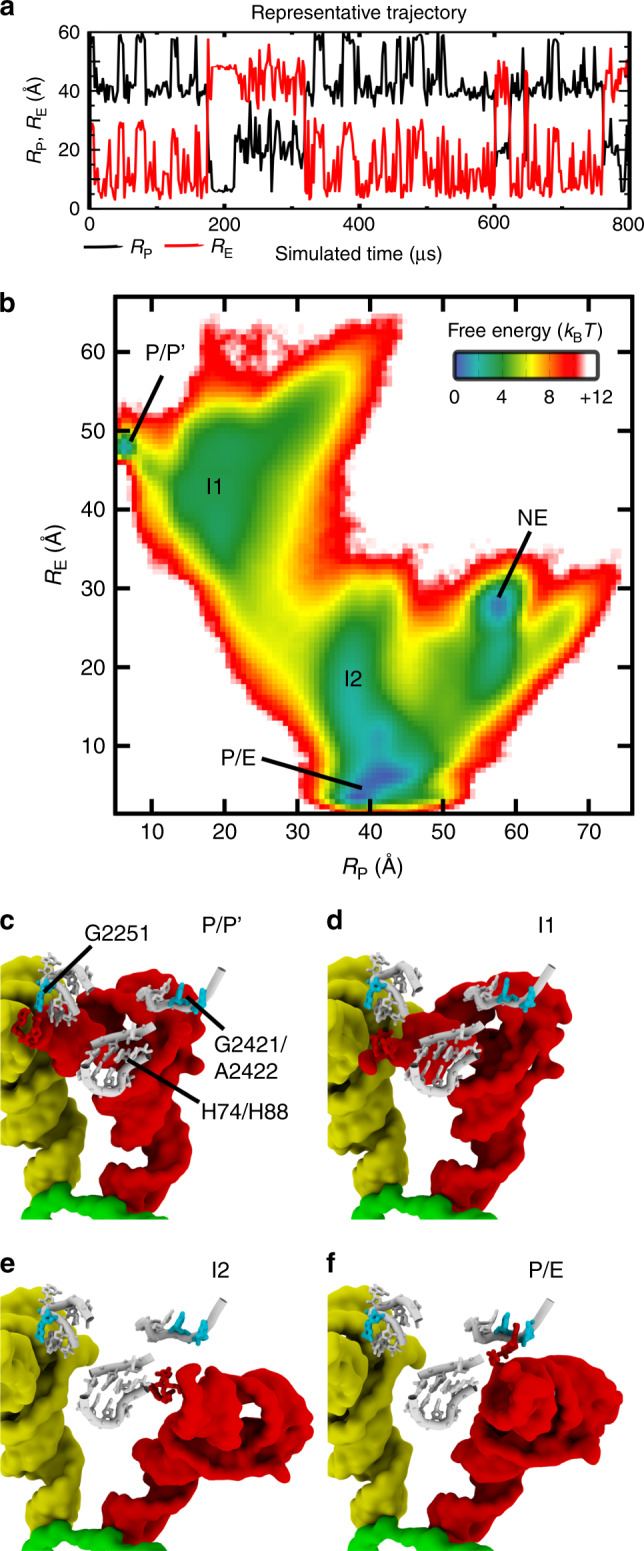
Capturing sterically-induced intermediates during P/E-hybrid formation. **a** Using an all-atom structure-based model^42,43^ of the ribosome, we simulated spontaneous rearrangements, in which the tRNA is displaced between the P and E sites of the large subunit. Transition events are apparent when monitoring the distance of the 3′-CCA end from the P site (*R*_P_; Supplementary Fig. 2) and E site (*R*_E_; Supplementary Fig. 3) of the large subunit. **b** The 2D free-energy *F*(*R*_P_, *R*_E_) reveals minima that correspond to the tRNA interacting with the P or E site (P/P' and P/E), as well as potential intermediates (I1, I2, and NE). Structural snapshots of the P/P' (panel **c**), I1 (panel **d**), I2 (panel **e**), and P/E (panel **f**) ensembles provide an overview of the simulated transitions. Analysis of microscopic rates indicates that I1 and I2 are obligatory intermediates, while NE is likely off-route. Additionally, since P/P′ is not sterically accessible once the 3′-CCA end of the A-site tRNA binds the P site, it will not be analyzed further. In the I1 and I2 ensembles, the tRNA is dissociated from the P site (G2251) and E site (G2421, A2422), where H74/H88 appears to spatially separate these intermediates. See Supplementary Note 1 for summary of structural nomenclature.

In the current study, we use energy landscape concepts and molecular simulations, in order to identify structural elements of the ribosome that can limit the kinetics of tRNA hybrid-state formation. Specifically, we evaluated the extent to which the steric composition of the ribosome contributes to the free-energy barriers that separate P/P and P/E conformations of tRNA (Fig. 1). To address this, we applied an all-atom structure-based (i.e. Gō-like) model^42^ to simulate spontaneous and reversible transitions of tRNA between the P and E sites of the large subunit (Fig. 2a). Despite the simplicity of the energetic model, where the pre-translocation and post-translocation configurations are explicitly defined to be stable, we find that the structure of the ribosome predisposes tRNA molecules to adopt distinct intermediates. Analysis of the associated transition state ensembles reveals the presence of a gate-like region on the large subunit that can control the rate-limiting step during hybrid rearrangements. Additional calculations demonstrate that, due to the position of the steric barrier, the kinetics of P/E formation only depends weakly on the position of the A-site tRNA. The existence of a pronounced sterically-induced barrier highlights the intimate relationship between biomolecular structure and kinetics, while also suggesting strategies for gaining precise control of ribosome dynamics.

## Results

### Simulating spontaneous and reversible P/E hybrid rearrangements

To determine whether specific structural features (e.g. helices/loops) of the ribosome are likely to regulate the kinetics of hybrid-state formation, we simulated the ribosome using an all-atom structure-based model^42,43^. With this model, we observed over 120 spontaneous transitions, where the tRNA molecule moves between the P and E sites of the large subunit, while the small subunit maintains a rotated orientation. Interconversion events were initially identified by measuring the distance of the 3′-CCA end of the tRNA from the two binding sites (*R*_P_, *R*_E_; see “Methods” section and Supplementary Figs. 2 and 3 for definitions). Large (~40 Å) anti-correlated changes in *R*_P_ and *R*_E_ (Fig. 2a) occur when the 3′-CCA end dissociates from the P (E) site and associates with the E (P) site. In addition, the tRNA elbow is displaced by  ~20 Å, as measured by *R*_FRET_ (distance between residues labeled in typical single-molecule experiments^27^; see “Methods” section and Supplementary Fig. 4 for definition). In contrast to previous simulations of P/E dynamics, which have applied sub-microsecond explicit-solvent simulations^44^, or have included artificial-targeting forces^45^, the current study describes complete and spontaneous transitions of tRNA between the P and E sites of the large subunit. Accordingly, the presented simulations provide an opportunity to directly connect specific tRNA–ribosome interactions with the energetics of large-scale barrier-crossing events.

In order to properly interpret the simulated dynamics, it is necessary to consider some features of the employed model (i.e. the force field, or potential energy function). First, by explicitly representing all non-hydrogen atoms, it is possible to identify free-energy barriers and intermediates that are induced by the steric composition of the ribosome^38^. Next, the endpoints of translocation (classical P/P and E/E configurations) are explicitly defined as potential energy minima. Since known structures define the force field, one typically refers to this representation as an all-atom "structure-based” model^42,43^. This type of model implicitly accounts for the cumulative effect of all (direct and indirect) interactions that stabilize the endpoints, such that it describes the effective energetics of a system^35,46,47^. Similar to studies of proteins^48^, A/P hybrid formation^49^ and tRNA–mRNA translocation^12^, the multi-basin character of the model is ensured by defining every bonded and non-bonded interaction to stabilize the pre-translocation or post-translocation configuration. While the positions of the minima are pre-defined, the depths are not intended to be precise. Accordingly, the free-energy difference between the endpoints, which is determined by the binding-site interaction strength, should be considered an adjustable parameter. Further, since the current simulations describe hybrid formation events when the rRNA is restrained to a rotated orientation, translocation of mRNA is not observed. However, previous simulations with a structure-based model, where the rRNA was not restrained, have been applied to describe the relationship between tRNA–mRNA translocation and rotation^12^. Finally, in the current implementation of the model, spontaneous A/P formation is precluded by defining all A-site tRNA interactions to stabilize the A/A conformation. This allows us to focus on the isolated movement of the P-site tRNA, which is consistent with experimental measurements that indicate P/E formation and A/P formation may occur sequentially^15,26,27^. In support of this simplification, we later show that the A-site tRNA position has a minimal effect on the implicated rate-limiting step during P/E formation. When considering the above features and limitations of the model, our immediate objective is to use the simulated events to characterize the roles of sterics and structural perturbations on the energy landscape of tRNA. Through this, we aim to identify structural "hot spots” that may be responsible for controlling the kinetics of P/E formation.

### Ribosome structure leads to multiple intermediates

We find that the structural characteristics of intermediates predicted by our model are consistent with various experimentally-identified features of hybrid formation. As inferred through biochemical measurements^8^, in simulations of an unrotated (*ϕ* ≈ 0°. Angle defined in ref. ^12^) ribosome the tRNA molecule remains bound to the P site of the large subunit. In this case, the tRNA–tRNA elbow distance (*R*_FRET_) is consistent with crystallographic structures of the A/A–P/P conformation (Supplementary Fig. 5). In contrast, when the rRNA is restrained to a rotated orientation (TI${\,}^{{\rm{pre}}}$ conformation of ref. ^10^, *ϕ* ~ 7°), the tRNA molecule spontaneously and reversibly transits between the P and E sites of the large subunit (Fig. 2a). The tRNA molecule is found to frequently sample the E site, while the P site is only rarely sampled, which highlights the significant influence of subunit rotation on tRNA position. The ability of tRNA to spontaneously adopt P/E-like configurations in the absence of EF-G was shown in early biochemical studies^7^, whereas the reversible character of the simulated transitions is consistent with a range of single-molecule measurements^19,27,50,51^. While these features of the simulated dynamics are similar to phenomenological observations in experiments, there is no clear consensus on the precise (energetic) relationship between intraribosomal rearrangements and tRNA motion. For example, while various single-molecule studies^27,50,51^ implicate a dependence of P/E formation on conformational events within the rotation, others have suggested tRNA molecules are not tightly coupled to ribosomal components^20^. To varying degrees, the presented results are consistent with both perspectives. That is, we find the P/E conformation is only accessed when the ribosome is rotated and that rotation disfavors tRNA binding of the P site. However, when the subunit is rotated, the flexibility of tRNA is sufficient to allow for reversible sampling of both the P and E sites of the large subunit. Even though tRNA flexibility could allow for P-site binding in a rotated ribosome, this is not expected to occur under typical elongation conditions, as this would likely be sterically occluded by an A/P-like tRNA molecule (see later discussion). Nonetheless, these observations suggest that the relationship between rotation and tRNA position may appropriately be described in terms of a population shift mechanism, where rotation favors the P/E ensemble.

Upon further comparison of the dynamics when the small subunit is either rotated, or unrotated, we find three dominant P-site tRNA elbow positions are accessible. The apparent population of three distinct tRNA–tRNA elbow distances is consistent with the single-molecule measurements of Munro et al.^27^, though it should be noted that other studies only reported two states^19,50,51^, suggesting the number of detected states may be influenced by various factors (e.g. buffer conditions, time resolution and/or the placement of the fluorophores). When three states were detected, they were interpreted as describing a  ~20 Å displacement of the P-site tRNA, followed by (or concomitant with) a  ~10 Å displacement of the A-site tRNA. In our simulations, when the ribosome is unrotated, the tRNA molecules maintain an A/A–P/P configuration, where the mean value of *R*_FRET_ is  ~43 Å (Supplementary Fig. 5). In contrast, when the small subunit is restrained to a rotated orientation, the distribution of *R*_FRET_ exhibits two pronounced peaks at 55 Å and 65 Å (Supplementary Fig. 6b). Thus, while the experiments were interpreted as signifying movement of both the A-site and P-site tRNA molecules, our analysis shows that three distinct ensembles may arise from isolated movement of the P-site tRNA. However, as discussed in later sections, the experimental signals are also compatible with rearrangements in both tRNA molecules.

In addition to corroborating inferences based on biochemical and single-molecule measurements, the simulations implicate intermediate conformations that are similar to cryoelectron microscopy (cryo-EM) reconstructions. That is, the two-dimensional free energy *F*(*R*_P_, *R*_E_) reveals the presence of possible intermediate minima that may be encountered during P/E formation (labeled I1, I2 and NE in Fig. 2b, see Supplementary Note 1 for summary of structural nomenclature). The I1 ensemble is composed of configurations in which the tRNA elbow and 3′-CCA end are dissociated from, but remain near, the ribosomal P site (Fig. 2d and Supplementary Fig. 7). In the I2 ensemble, the tRNA elbow adopts a P/E-like position (Supplementary Fig. 7), while the 3′-CCA end samples configurations proximal to the E site (Fig. 2e). For completeness, we will note that there is also weak sampling of P/P-like configurations (labeled P/P′). This ensemble is minimally sampled when the ribosome is rotated, and it is only accessible when the A-site tRNA is in an A/A conformation. However, displacement of the 3′-CCA end of the A-site tRNA to the P site (G2251) would result in a steric clash with the P/P′ ensemble configurations. Since A-site tRNA 3′-CCA end movement is likely associated with peptide bond formation, and therefore release of the P site by the P-site tRNA, P/P′ configurations are not expected to be realized during elongation. Accordingly, we will not consider the P/P′ ensemble in subsequent analysis. While we exclude P/P′ from further consideration, the apparent intermediates (I1 and I2) suggest hybrid formation involves initial dissociation of the 3′-CCA end from the P site, followed by a large-scale displacement of the tRNA elbow and arm, and then binding to the E site. This sequence of events is qualitatively consistent with the cryo-EM study of reverse translocation by Fischer et al.^15^. Specifically, our I1 ensemble is similar to a structural model of the Pre-2 configuration^44^, where the 3′-CCA end is dissociated from the P site and the elbow is displaced in the direction of the E site, while the acceptor arm is near A2255 (G2255 in TT; Supplementary Fig. 8). In addition, I2 is similar to a structural model of the Pre-3 configuration^44^, where the elbow and arm are in P/E-like positions, while the 3′-CCA tail is near the E site (Supplementary Fig. 9). Finally, the Pre-4 structure and P/E ensemble correspond to conventional hybrid configurations, where the 3′-CCA end is interacting with the E site. Our model also implicates the presence of an additional free-energy minimum (NE), where the tRNA elbow is in a P/E-like position (Supplementary Fig. 7) and the 3′-CCA end is distal to both the P and E sites (Supplementary Fig. 10). However, as described below, analysis of the kinetics suggests the NE ensemble is typically reached after P/E formation has completed.

Interestingly, cryo-EM models of the ribosome in complex with release factors (RF) have revealed a tRNA conformation (Structure III^52,53^) that is similar to our identified I1 ensemble (Supplementary Fig. 11). While the I1 conformation is shifted, due to a higher degree of head and body rotation than in the RF models, C72 and A73 of all three tRNAs are positioned near G2255 of the 23S rRNA. Since the molecular components of these systems differ (tRNA^fMet^ with RF vs. tRNA^Phe^ in simulations), the similar positioning of the tRNA molecules is consistent with ribosomal sterics being a major determinant of tRNA conformation. Taken together, the range of similarities between the simulated dynamics and single-molecule/cryo-EM measurements suggests that a minimal set of physical properties can give rise to the complex dynamics that have been observed in experiments.

### Rate-limiting free-energy barrier is sterically induced

A motivation for applying energy landscape techniques is to identify the molecular factors that govern biological dynamics. To provide such insights for P/E formation, we first determined which conformational substep is rate-limiting in the simulations and then compared the associated rates with experimental measurements. As described below, this analysis provides evidence that steric interactions likely govern the transition state and kinetics of P/E formation.

**Fig. 3:**
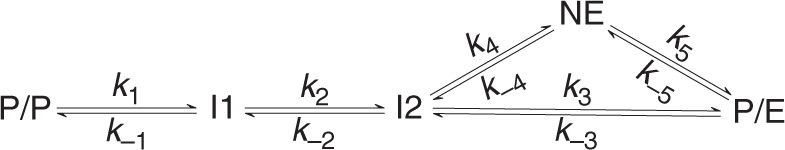
Kinetic scheme implicated by simulations. Transitions between I1 and P/E may occur via I2, or I2 and NE.

To identify the rate-limiting step in the simulations, we calculated the relative rates of interconversion between the I1, I2, P/E, and NE ensembles (see “Methods” section and Supplementary Fig. 12 for definitions). As described above, since the P/P′ ensemble is not expected to be accessible under typical elongation conditions, it will not be analyzed further. That is, while this configuration is occasionally sampled when the A-site tRNA is in an A/A conformation, it will be sterically occluded once the 3′-CCA end of the A-site tRNA binds the P site (G2251) of the large subunit. In the simulations, there were numerous (>20) spontaneous (and reversible) transitions between the following ensembles: I1:I2, I2:P/E, I2:NE, and NE:P/E (Supplementary Table 1). These considerations suggest the kinetic model shown in Fig. 3 for describing the dynamics. In this kinetic scheme, “P/P” signifies a classical tRNA in an unrotated ribosome. Thus, the effective rate *k*_1_ represents the initiation of P/E formation, where there is a combination of subunit rotation and release of the P-site tRNA from the P site. However, since the current simulation did not directly study the rotation process, *k*_1_ cannot be extracted from the current data set. After P/E formation is initiated and the tRNA has reached the I1 ensemble, it may proceed directly from I2 to P/E, or pass through the NE intermediate. While NE could represent an en-route intermediate, we find that *k*_3_ ≈ 1000*k*_4_, suggesting the NE path is not kinetically competitive. In addition, *k*_−5_ ≈ 15*k*_4_, indicating that it is more probable for tRNA to adopt the P/E configuration prior to reaching the NE ensemble. Since the NE ensemble is either off-route, or it is sampled after the completion of hybrid-formation, we conclude that the I1–I2 transition (*k*_2_) is the rate-limiting step in the simulations, where ${k}_{2}\approx \frac{{k}_{3}}{200}$.

**Fig. 4:**
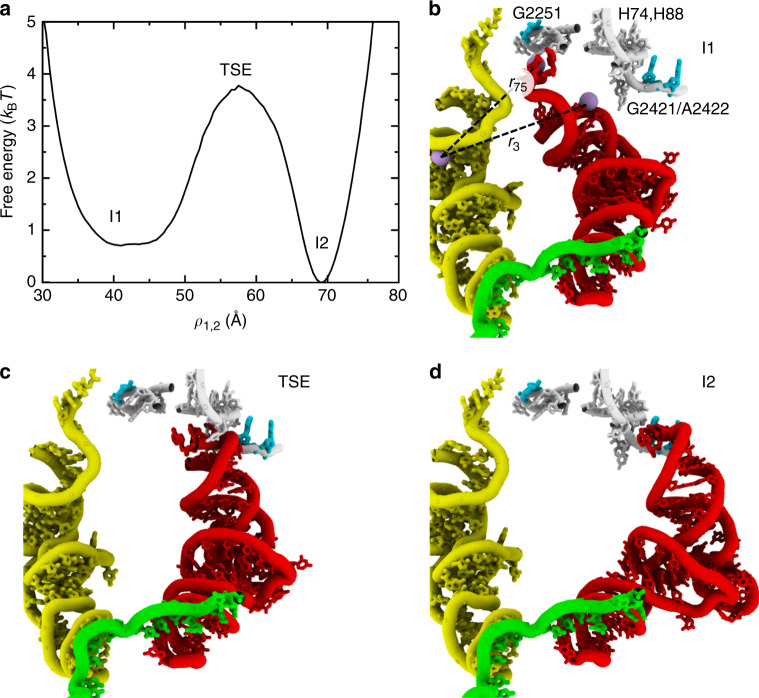
Identifying the rate-limiting free-energy barrier. **a** Since the I1–I2 transition is rate-limiting in the simulations (Supplementary Table 1), we calculated the free-energy as a function of the tRNA position for this step. To specifically describe the I1–I2 transition, the free energy was calculated for simulated frames that were within these ensembles and the associated transition state ensemble (TSE). The I1–TSE–I2 configuration space was defined as 12 Å < *R*_P_ < 42 Å and 15 Å < *R*_E_ < 50 Å. The coordinate *ρ*_1,2_ was used for analysis, after applying transition path analysis to thousands of possible tRNA coordinates. *ρ*_1,2_ is defined as a linear combination of two interatomic distances that monitor the 3′-CCA end (*r*_75_) and the acceptor arm (*r*_3_). *r*_*i*_ is the distance between O3′ atoms of residue *i* of the P-site tRNA and residue 67 of the A-site tRNA. **b** Structural snapshot of the I1 ensemble (*ρ*_1,2_ ~ 40 Å), rotated relative to Fig. 2. **c** Representative snapshot of the transition-state ensemble (*ρ*_1,2_ ~ 57 Å). Visual inspection would suggest the TSE is characterized by close interactions between the tRNA and H74/H88. **d** Representative I2 configuration (*ρ*_1,2_ ~ 70 Å).

Consistent with the slow rates of interconversion between the I1 and I2 intermediates, we find that the steric composition of the ribosome introduces a pronounced free-energy barrier (Fig. 4). Since structurally-inspired reaction coordinates for the ribosome typically provide inaccurate measures of free-energy barriers and transition states^54,55^, we applied transition path (TP) analysis^56,57^ to identify a coordinate that is suitable for studying the I1–I2 transition (see “Methods” section for details). Specifically, we used *ρ*_1,2_, which is defined as a linear combination of two interatomic distances: 0.4*r*_3_ + 0.6*r*_75_. *r*_*i*_ is the distance between O3′ atoms of residue *i* in the P-site tRNA and C67 in the A-site tRNA (Fig. 4b). 123 interconversion events were detected along *ρ*_1,2_ and the calculated free-energy barrier was 3–4*k*_B_*T* (Fig. 4a). Free-energy barriers were recalculated using 1/5 of the simulated data to verify robustness (Supplementary Fig. 13).

We next asked whether the apparent free-energy barrier arises from steric effects, or from the competition of stabilizing interactions with the P and E sites of the large subunit. For this, we calculated the number of stabilizing P-site or E-site interactions that were formed, as functions of the tRNA position. A contact was defined as being "formed” if the atom pair is within 1.2 times the distance found in the respective endpoint conformation (P/P or E/E). In the TSE region (*ρ*_1,2_ ≈ 57 Å), nearly every P-site and E-site contact is broken. Specifically, the probability of forming any given stabilizing E-site contact is <0.02, except for one contact that has a probability of 0.047. Similarly, the probability of forming a stabilizing P-site contact is <0.02 for all interactions, except two contacts that have probabilities of 0.039 and 0.57. The single high-probability contact is with H69, and it maintains a probability of about 0.5 when in the P/P and P/E ensembles. Accordingly, the general lack of energetically stabilizing contacts in the TSE indicates that the barrier separating the I1 and I2 ensembles may be directly attributed to steric interactions between the tRNA molecule and ribosome. With regards to model parameters, this indicates that an increase in the strength of P/P and E/E stabilizing contacts would reduce the free energies of the I1 and I2 ensembles, while leaving the TSE minimally affected. Since the binding site contacts are more accessible in the I1/I2 ensembles than the TSE, changes in their strength would primarily alter the intermediate ensembles. Accordingly, increasing affinity of tRNA for the ribosomal-binding sites would amplify the existing sterically-induced free-energy barrier. Based on these considerations, the kinetics of the I1-to-I2 transition implicated by our sterics-centric model should be considered a lower-bound.

**Fig. 5:**
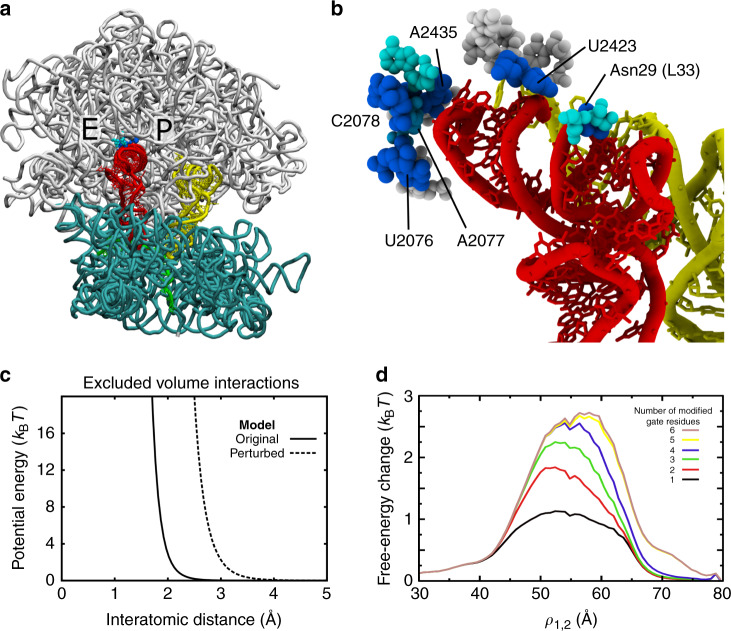
A steric gate limits P/E kinetics. **a** Representative snapshot of the TSE associated with the I1–I2 transition (proteins not shown). Residues that specifically contact the tRNA in the TSE are shown in cyan and blue. **b** Close-up perspective of a TSE configuration, where spheres indicate which residues contact tRNA in the TSE (*p* > 0.1). Blue indicates that Δ*p*_i_ > 0.35: i.e. the probability of forming a contact is greater for the TSE than I1, or I2, by at least 0.35. Interactions with six residues were found to be specific to the TSE: U2076, A2077, C2078, A2435, U2423, and Asn29 of protein L33. **c** To test whether these six residues strictly limit barrier-crossing events, we calculated perturbed free-energy profiles, where the excluded volume of individual residues was increased from 2.1 Å (original model) to 3.0 Å (perturbed model). Panel shows the functional form of the original and perturbed interactions. **d** Increase in free-energy, as a function of *ρ*_1,2_, when different numbers of residues (*N* = 1–6) were perturbed. All possible combinations of gate residues were tested. For each value of *N*, the combination of residues that yielded the largest increase is shown. The most significant increase in barrier is found when all six residues are perturbed. However, comparable changes are found when as few as four residues are perturbed (U2076, U2423, A2435, Asn29), suggesting these residues represent the minimal constituents required to limit P/E formation.

Consistent with experimentally measured rates, we find that our sterics-centric model implicates millisecond timescales for P/E formation. Using a barrier-crossing attempt frequency estimated from explicit-solvent simulations, the calculated free-energy barrier corresponds to a crossing time of  ~0.2–0.5 ms. Specifically, Fig. 5 (panel f) of ref. ^58^ estimates the prefactor as  ≈0.1–0.2/μs for a barrier of 4*k*_B_*T*, which corresponds to a timescale of  ~0.5 ms. This is consistent with explicit-solvent simulations^44^ that have predicted transitions between I1 (Pre-2) and I2 (Pre-3) occur on timescales greater than microseconds. Experimentally, rapid kinetic assays have also estimated the timescale of transitions between P/P and P/E conformations to be ~30 ms^26^. One may partially reconcile the longer experimental timescale by noting that the calculated timescale is likely underestimated by a factor of  ≈2, even though TP analysis was applied^54^. In addition, as described in the next section, sub-Å changes to ribosome sterics can decrease the rate by a factor of 12–15. Thus, minor structural fluctuations that may arise from experimental conditions (e.g. buffer, temperature) could account for an order-of-magnitude difference in rates. Together, these two considerations suggest the sterically-induced barrier imposes a lower-bound on the timescale of  ≈10 ms. As a note, single-molecule experiments typically report longer timescales (200 ms–1 s)^19,27,50,51^, suggesting experiment-specific factors may further amplify the sterically-induced barrier by several *k*_B_*T*. To ask whether the lack of electrostatic interactions in our model also contributes to the differences with experimental rates, we used a free-energy perturbation approach (see “Methods” section) to calculate the free energy when screened electrostatic interactions (Debye–Hückel) are included. We find that screened electrostatic do not amplify the barrier height (Supplementary Fig. 14), though a more-detailed representation would be required to fully characterize the influence of electrostatic interactions. In summary, while other conformational processes (e.g. subunit rotation and release of the P site) may also occur on relatively slow timescales, the presented calculations reveal how steric interactions, alone, can ensure that P/E formation occurs in the millisecond regime.

### Steric "gate” controls tRNA rearrangements

Since the sterics-centric model and experimental measures both implicate millisecond timescales, detailed structural analysis of the simulated events may suggest which interactions limit the kinetics of P/E formation on the ribosome. To identify interactions that are specific to the transition state (Fig. 4a), we calculated the probability that each residue in the large subunit makes a steric contact with the tRNA (defined by a cutoff distance of 4 Å). For notation, ${p}_{{i}}^{{j}}$ denotes the probability that residue *i* of the ribosome is in contact with the tRNA in ensemble *j* (TSE defined as 56.3 Å < *ρ*_1,2_ < 58.3 Å; I1, 41 Å < *ρ*_1,2_ < 41.1 Å; I2, 72.09 Å < *ρ*_1,2_ < 72.10 Å). We then evaluated $\Delta {p}_{{\rm{i}}}={p}_{{\rm{i}}}^{{\rm{TSE}}}-\max ({p}_{{\rm{i}}}^{{\rm{I}}1},{p}_{{\rm{i}}}^{{\rm{I}}2})$. Positive values indicate the tRNA molecule is more likely to contact residue *i* in the TSE than in either of the endpoints. As expected, Δ*p*_i_ ≤ 0 for residues near the P and E sites. However, there are 14 residues for which Δ*p*_i_ > 0.1. Of these, Δ*p*_i_ > 0.35 for six: U2076, A2077, C2078, U2423, and A2435 of the 23S rRNA, and Asn29 of protein L33. Structurally, these residues appear to introduce a narrow passageway through which the tRNA molecule must transit in order to adopt the P/E configuration (Fig. 5a, b). While non-equilibrium targeted MD simulations have suggested the tRNA can interact with many (~100) residues in the large subunit during P/E formation^45^, the current analysis directly correlates formation of a small set of tRNA–ribosome contacts with the rate-limiting free-energy barrier.

Sub-angstrom changes to the steric gate can lead to an order-of-magnitude change in kinetics. To demonstrate this, we calculated the change in free energy when the excluded volume of individual residues is increased. Consistent with previous applications of this model^12,49,54,59,60^, the excluded volume between atoms is given by ${V}_{{\rm{ex}}}=\epsilon {\left(\frac{\sigma }{r}\right)}^{12}$, where *ϵ* = 2*k*_B_*T* and *σ* = 2.1 Å (Fig. 5c). For free-energy perturbation calculations, we considered an increase in the excluded volume interaction (*σ* = 3.0 Å) between the tRNA and individual gate residues (U2076, A2077, C2078, U2423, A2435, and Asn29). We also calculated the change in free energy for all possible combinations of steric-gate residues. When the interactions with all six gate residues are perturbed, the free-energy barrier is increased by 2.5–2.7*k*_B_*T* (Fig. 5d), which corresponds to a decrease in the rate by a factor of 12–15. However, similar increases in the barrier are also obtained when only five (U2076, C2078, U2423, A2435, Asn29) or four (U2076, U2423, A2435, Asn29) residues were perturbed (Fig. 5d). When fewer than four residues are modified, the effect on the barrier height is largely attenuated. These observations indicate that the minimal constituents of the steric gate are U2076, U2423, A2435, and Asn29. Together, this set of residues introduces stringent limits on the accessible tRNA pathways during P/E formation, such that small changes in their relative positions can have dramatic effects on the kinetics. From an experimental standpoint, the presence of this constriction suggests that it may be possible to modulate P/E kinetics by mutating residues in, or near, the gate. For example, introducing a larger side chain (or insertion) at position 29 of L33 would be expected to amplify the steric composition of the gate-like region, and thereby reduce the rate of P/E formation. Similarly, it may be possible to introduce pyrimidine-to-purine substitutions in the gate-like rRNA residues, in order to contract this narrow passageway in experiments.

### Influence of the steric gate is robust to A-site tRNA position

**Fig. 6:**
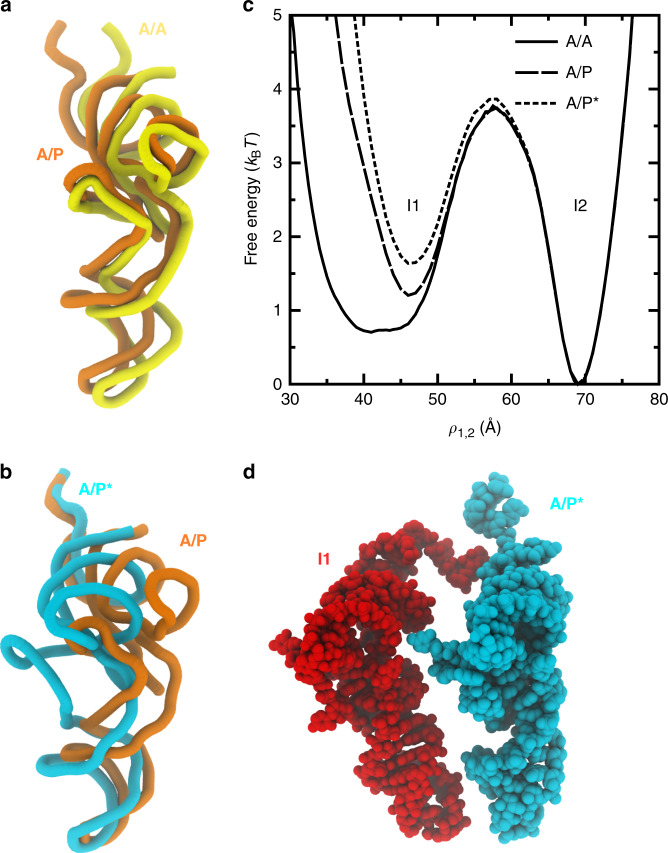
Intermediates during P/E formation are robust to A-site tRNA position. **a** Tube representation of A/A (yellow) and A/P (orange; PDB: 4V7C^9^) tRNA positions. Structures are alignment based on the 23S rRNA atoms. **b** Tube representation of the A/P and A/P* (light blue; PDB: 4V7D^9^) structures. **c** Free-energy profiles reveal that the barrier separating the I1 and I2 ensembles is marginally influenced by the A-site tRNA position. **d** A structural snapshot illustrates how the ribosome can simultaneously accommodate tRNA molecules in the I1 (red) and A/P* (light blue) conformations.

To elucidate the possible interplay between A-site and P-site tRNA dynamics, we asked whether the predicted P/E intermediates (I1 and I2) and the associated transition state ensemble will depend on the position of the A-site tRNA. Since the simulations were constructed to probe transitions of a single tRNA between the P and E sites of the large subunit, we utilized a perturbative approach to analyze the influence of the A-site tRNA. Specifically, we aligned cryo-EM structures of ribosomes with A/P and A/P* tRNA molecules (PDB ID: 4V7C and 4V7D^9^, see Supplementary Note 1 for complete list of terminology/notation) to the simulated system (Fig. 6a, b). We then identified all simulated frames for which the A/P or A/P* tRNA would introduce a steric clash (two atoms within 2.5 Å) with the simulated tRNA. Frames that would encounter a steric clash were removed from the data set and the free energy was re-calculated according to $F=-{k}_{{\mathrm{{B}}}}T\mathrm{ln}\,(P)$. This is equivalent to performing a free-energy perturbation calculation (Eq. (4)) with a hard-sphere potential for each atom in the A-site tRNA molecule.

When the sterics of an A/P or A/P* tRNA molecule are accounted for, there is a minimal increase in the free energy of the I1 ensemble, and the pronounced barrier separating the I1 and I2 ensembles remains clearly visible (Fig. 6c). This small increase in the free energy of the I1 ensemble may be attributed to steric confinement of the P-site tRNA, similar to the predicted effects of EF-Tu^59^ and the L11 stalk^60^ during accommodation, or to the influence of molecular crowders on protein stability^61^. However, the minimal effect of the A-site tRNA position on the I1–I2 transition reveals that the I1 ensemble is structurally compatible with A/P and A/P* tRNA conformations (Fig. 6d). That is, since the I1 ensemble is associated with a P-site tRNA molecule that is dissociated from the P site of the large subunit, there is sufficient space for the A-site tRNA to simultaneously adopt an A/P or A/P* configuration. Accordingly, the I1–I2 transition is likely to be associated with a pronounced sterically-induced free-energy barrier, which will not strongly depend on the A-site tRNA position.

The ability of the ribosome to simultaneously accommodate the I1 ensemble and various A-site tRNA configurations has potential implications in the interpretation and development of single-molecule experiments. For example, Munro et al.^27^ reported three distinct FRET states (high, mid, and low FRET), which were assigned A/A–P/P, A/P*–P/E, and A/A–P/E (C, H1, and H2, Munro nomenclature) conformations. While this interpretation was motivated by contemporary structural models, our analysis indicates that the employed FRET probes have the potential to mask several sterically-accessible states/ensembles during hybrid formation. That is, there may be additional “hidden” conformations that yield indistinguishable FRET signals. For example, in the presented calculations, the distribution of *R*_FRET_ values for the A/P*–I1 ensemble is peaked at 42.5 Å, while the value obtained for the A/A–P/P structure^63^ is 42.1 Å (Supplementary Fig. 15). Next, the distribution of *R*_FRET_ values for the A/P–I1 ensemble is peaked at  ~53 Å, whereas the value found in the A/P*–P/E structure is 53.3 Å (Supplementary Fig. 16). Finally, given the similar positioning of the tRNA elbow in the I2 and P/E ensembles, these two conformations are likely to report very similar FRET signals (Supplementary Fig. 7). Thus, while previously employed FRET probes identified three states, we show that these signals are consistent with seven distinct combinations of A-site and P-site tRNA conformations (A/A with P/P, A/P with I1, I2 or P/E, as well as A/P* with I1, I2, or P/E). This raises the prospect that hybrid formation may be associated with numerous parallel pathways, where novel biochemical and spectroscopic techniques (e.g. multi-color FRET) will likely be necessary to unambiguously distinguish between this expanded set of possibilities.

### Aspects of the gate-like region are bacteria-specific

Sequence analysis suggests that the gate-like region may represent a viable therapeutic target, where the excluded volume of a bound small molecule could be sufficient to halt tRNA motion in bacteria. In order to be an effective target, this region should differ in bacterial and human (cytosolic and mitochondrial) ribosomes. Recent phylogenetic analysis of rRNA sequences across kingdoms of life showed that only A2423 is positioned within a moderately variable region, in terms of structure and sequence (see Fig. 2 of ref. ^62^). While the other rRNA gate residues are largely conserved, the remaining gate residue, Asn29 in protein L33, is distinct from its counterparts in cytosolic and mitochondrial human ribosomes. Structure alignment (Supplementary Fig. 17a) shows that L33 binds the same position in bacteria and mitochondria, and protein L36A binds a comparable position in the human cytosolic ribosome. However, the position of Asn29 in bacteria coincides with Leu37 in mitochondria, and there is not a corresponding residue at this position in L36A (Supplementary Fig. 17b, c). In addition, the immediately downstream sequence in bacteria (*T. thermophilus*) is Thr–Pro–Asn, while in human mitochondria it is Arg–Glu–Lys. While the backbone orientations are similar in this region, it is possible that one may be able exploit these differences in sequence to specifically target bacterial elongation kinetics.

In addition to exhibiting differences between L33 and L36A, the sequence around Asn29 is relatively conserved across bacterial species (Supplementary Fig. 18). Further, the corresponding residues in mitochondrial L33 are infrequently found in bacteria. Sequence alignment of 840 L33 proteins (Supplementary Methods) reveals that position 29 (*T. thermophilus* numbering) is found to be Asn in most organisms (51.5%), while the second most common residue is Thr (28.9%). Position 28 also exhibits minimal variations, where most bacteria contain Arg (66.0%) or Lys (22.3%). Position 30 was slightly more variable, where Thr (21.3%), Met (20.7%), Lys (14.0%), Asn (13.2%) and Asp (11.4%) were most common. Positions 31 and 32 were also relatively conserved, where the two most common residues at each position account for  ~80% of bacterial sequences. At position 31, Pro (63.9%) and Thr (22.5%) were most common, while Glu (50.2%) and Asp (27.0%) were most frequent in position 32. Interestingly, Trp and Phe were the only residues not found within positions 28–30 for any organism, and Tyr was only found within this region in a single organism. The near-complete lack of larger residues would be expected, based on the presence of a steric gate centered at position 29. Since sub-Å changes to the gate can lead to an order-of-magnitude change in the rate, large side chains at this position would have the potential to severely impede elongation dynamics.

Overall, our analysis suggests L33 may be a suitable target for novel broad-spectrum antibiotics. That is, we find that the gate-like region satisfies some minimal requirements for such an application. First, occluding the gate-like region should impede elongation. Second, features of this region are specific to bacteria. Third, these features are common across bacterial species. While many additional factors must be considered when developing therapeutics, the current study provides a physical framework for the pursuit of a specific target that has the potential to influence elongation.

## Discussion

With continual increases in computing capacity, the development of novel theoretical models and advances in experimental techniques, the ribosome is becoming a model system for establishing general principles of biomolecular dynamics. To this end, we have focused on elucidating the influence of steric interactions on a large-scale conformational rearrangement that is essential for protein synthesis. By employing all-atom models that include a simplified description of the energetics, our calculations demonstrate how the steric composition of the ribosome imposes strict limitations on tRNA dynamics. While a complete description of the factors that control function will require models with more complete energetic representations, the current study illustrates how precisely introduced (sub-Å) perturbations to ribosome structure are sufficient to control large-scale dynamics. Specifically, our calculations demonstrate that a six-residue gate-like region can act as a modulator of hybrid-formation kinetics. This provides a concrete physical example of how ribosome structure dictates tRNA dynamics. In future studies, it will be interesting to determine whether the same structural features limit the kinetics in other organisms, and whether cellular conditions (ionic concentration, temperature, presence of co-factors/small molecules) can amplify/attenuate these structural signatures. In such endeavors, the current study will provide a quantitative foundation, upon which precise insights into complex dynamics may be obtained.

## Methods

### Structure-based model

All simulations employed an all-atom structure-based force field^42^, where the potential energy of the ribosome, mRNA, and tRNA molecules is defined to have minima based on the pre-translocation (PRE) and post-translocation (POST) configurations. In the PRE configuration, the ribosome is unrotated and the tRNA molecules are in the A/A–P/P conformation. The POST conformation consists of an unrotated ribosome and tRNAs in a P/P–E/E arrangement. The endpoint structures were based on a crystallographic structure of a classical ribosome with tRNA molecules in the A/A, P/P, and E/E conformations (PDB 4V6F^63^).

The multi-basin structure-based model was defined in several steps. First, separate single-basin all-atom structure-based models were generated for the PRE and POST complexes using the SMOG 2 software package^43^. In the multi-basin model, stabilizing interactions (bonds, angles, dihedrals, and contacts) corresponding to the PRE structure were assigned to the ribosome, the A-site tRNA, P-site tRNA, and mRNA molecules. We then introduced stabilizing contacts between the P-site tRNA and the E site. All tRNA–ribosome contacts defined by the POST model were included. According to this construction, the model for the P-site tRNA has multiple potential energy minima, where contacts between tRNA and both binding sites can compete to stabilize the P/P and E/E (or P/E) configurations. Various models with different weights for the P/P and E/E contacts were tested in order to produce spontaneous and reversible interconversion events between the binding sites, when the ribosome was rotated. Once parameters were selected, unrestrained simulations were performed, which showed that P/E formation is absent when the ribosome adopts an unrotated configuration (Supplementary Fig. 5).

The all-atom structure-based model explicitly represents every non-hydrogen atom. Harmonic potentials are used to preserve the backbone geometry (bond lengths, angles and improper/planar dihedral angles), while flexible dihedral angles are represented by cosine functions. Non-bonded atoms that are in contact in the PRE and POST structures are given 6–12 interactions with minima defined based on the endpoint configurations. These contacts were identified through use of the Shadow Contact Map algorithm^64^ with a cutoff distance of 6 Å, shadowing radius of 1 Å and residue sequence separations of 3 for proteins and 1 for RNA molecules. Atom pairs that are not identified as contacts are assigned an excluded volume interaction. The potential energy is given by

1 $$V 	= \mathop{\sum}\limits_{\,\text{bonds}\,}\frac{{\epsilon }_{{\rm{b}}}}{2}{(r-{r}_{{\rm{o}}})}^{2}+\mathop{\sum} \limits_{\,\text{angles}\,}\frac{{\epsilon }_{{{\theta }}}}{2}{(\theta -{\theta }_{{\rm{o}}})}^{2}+\ \mathop{\sum}\limits_{\,\text{impropers/planar}\,}\ \frac{{\epsilon }_{{\rm{\chi }}}}{2}{(\chi -{\chi }_{{\rm{o}}})}^{2}\\ 	\quad+\mathop{\sum} \limits_{\,\text{backbone-dihedrals}\,}{\epsilon }_{{\rm{bb}}}\ F(\phi )+\mathop{\sum}\limits_{\,\text{sidechain-dihedrals}\,}{\epsilon }_{{\rm{sc}}}\ F(\phi )\\ 	\quad+\mathop{\sum}\limits _{\,\text{non-contacts}\,}\ {\epsilon }_{{\rm{nc}}}{\left(\frac{{\sigma }_{{\rm{nc}}}}{{r}_{ij}}\right)}^{12}+\mathop{\sum}\limits_{\,\text{contacts}\,}{\epsilon }_{{{\rm{c}}}_{{\rm{k}}}}\ f({\sigma }_{ij,k},{r}_{ij})$$

where

2 $$F(\phi )=\left[1-\cos (\phi -{\phi }_{{\rm{o}}})\right]+\frac{1}{2}\left[1-\cos (3(\phi -{\phi }_{{\rm{o}}}))\right]$$

and the contacts function is given by

3 $$f(\sigma ,r)={\left(\frac{\sigma }{r}\right)}^{12}-2{\left(\frac{\sigma }{r}\right)}^{6}.$$

$\left\{{r}_{{\rm{o}}}\right\}$, $\left\{{\theta }_{{\rm{o}}}\right\}$, $\left\{{\chi }_{{\rm{o}}}\right\}$ and $\left\{{\phi }_{{\rm{o}}}\right\}$ were given the values found in the A/A–P/P configuration. *ϵ*_b_ = 100ϵ/Å^2^, *ϵ*_*θ*_ = 80*ϵ*/rad^2^, *ϵ*_*χ*_ = 40*ϵ*/rad^2^, *ϵ*_nc_ = 0.1*ϵ*, and *σ*_nc_ = 2.5 Å, and the dihedral-contact ratio was normalized, consistent with previous implementations of this model^43^. As noted above, contacts were defined based on two atomic structures: the A/A–P/P (PRE) and P/P–E/E (POST), each with native distances $\left\{{\sigma }_{ij,{\rm{PRE}}}\right\}$ and $\left\{{\sigma }_{ij,{\rm{POST}}}\right\}$. In our model, we rescaled $\left\{{\sigma }_{ij,{\mathrm{{PRE}}}}\right\}$ by 0.96 to prevent artificial inflation due to entropic effects. According to the default calculation of contact strength^43^, the strength of each contact was *ϵ*_c_ = 0.4951*ϵ*. However, since this model describes effective energetics, it was necessary to rescale the strength of tRNA–ribosome contacts (which form transiently), relative to the intra-ribosome contacts (which ensure structural stability of the assembly). After a parameter sweep, tRNA–50S contacts that are unique to the A/A–P/P configuration were given a weight of 0.15*ϵ*_c_, tRNA–50S contacts that are unique to the P/P–E/E configuration were given a weight of 0.275*ϵ*_c_. To ensure that the 3′-CCA end can stably bind the E site of the large subunit, contacts between A76 of the tRNA and residues G2421/A2422 were given a stronger weight of 1.5*ϵ*_c_.

### Simulation details

All force field files were generated using the SMOG 2 software package (smog-server.org)^43^. Molecular dynamics simulations were performed using Gromacs (v5.1.4)^65,66^. Simulations were performed at a reduced temperature of 0.5$\frac{\epsilon }{{k}_{B}}$, where the temperature was maintained through use of Langevin dynamics protocols. Reduced units were used for all calculations. To study P/E formation, 10 independent simulations were performed for a minimum of 7 × 10^8^ time steps (size 0.002), each. In order to probe the dynamics of tRNA when the ribosomal subunits are rotated, every ribosomal RNA atom (except for those in the L1 stalk) was harmonically restrained to a rotated conformation (TI${\,}^{{\rm{Pre}}}$ model, based on the cryo-EM study of ref. ^10^) with a spring constant of *k* = 0.1*ϵ*/Å^2^. We also performed one additional simulation without position restraints, in order to verify that the P-site tRNA remains in a P/P configuration when the ribosome is unrotated. While the reported rates are based on free-energy barriers, when using this model and simulation protocols to study tRNA motion inside of the ribosome, one may alternately estimate that one reduced unit corresponds to ~1 ns^67^. This factor is used to obtain the effective simulated timescales in Fig. 2a Supplementary Fig. 5.

### Definitions of reaction coordinates

To describe the dynamics of P/E formation, we employed the following structural metrics:

- *R*_P_—distance between the geometric centers of the C75 functional group (non-backbone) in the P-site tRNA and the functional group of G2251 (Supplementary Fig. 2).
- *R*_E_—distance between the geometric centers of the C76 functional group in the P-site tRNA and the functional groups of G2421 and A2422 (Supplementary Fig. 3).
- *R*_FRET_—distance between the geometric centers of U47 in the A-site tRNA and U8 in the P-site tRNA (Supplementary Fig. 4).
- *ρ*_1,2_—linear combination two interatomic distances: 0.4*r*_3_ + 0.6*r*_75_, where *r*_*i*_ is the distance between O3′ atoms of residue *i* in the P-site tRNA and C67 in the A-site tRNA (Fig. 4b). *ρ*_1,2_ was identified after an extensive analysis of possible collective coordinates (see below).

### Reaction coordinate analysis

In the diffusive regime, upon reaching the top of a free-energy barrier, the system will be equally likely to proceed to either the "reactant” or "product” state. That is, there is a probability of 0.5 that the system will successfully cross the underlying barrier. This may be expressed in terms of the conditional probability P(TP∣***x***): the probability that the system is undergoing a barrier-crossing event (i.e. on a Transition Path), given a configuration *x*. If *x* is within the TSE, then P(TP∣***x***) = 0.5. Similarly, if the coordinate *ρ* unambiguously identifies TSE configurations, then P(TP∣*ρ*) will also yield a value of 0.5 at the TSE^56,57^. Here, for the I1–I2 transition, we calculated P(TP∣*ρ*) for thousands of possible coordinates that are based on linear combinations of interatomic distances between the tRNA molecules. We find that a linear combination of two interatomic distances can minimize the number of apparent transitions (i.e. no false positives), while also yielding a value of P(TP∣*ρ*) ~ 0.5 (Supplementary Fig. 19). The highest-performing coordinate was *ρ*_1,2_ (defined above).

### Calculating microscopic rates

To calculate the microscopic rates between dominant free-energy minima, we first defined the ensemble/states in terms of *R*_P_ and *R*_E_. The I1, I2, P/E, and NE ensembles were defined as being centered at (*R*_P_, *R*_E_) = (18.5, 42.5), (37.5, 18.5), (37.5, 3.5), (57.5, 28). Values are given in units of Å. The system was defined as being in a state if $\sqrt{\Delta {R}_{\mathrm{{{P}}}}^{2}+\Delta {R}_{\mathrm{{{E}}}}^{2}}\, < \, 3\AA$, where Δ*R*_P/E_ is the difference between the instantaneous value of *R*_P/E_ and the center of the defined state (Supplementary Fig. 12). Once the system reached a state, the system was considered to be in that state, until the boundary of a different state was crossed. The microscopic rate between ensemble *i* and *j* was then defined as *k*_*ij*_ = *N*_*ij*_/*τ*_*i*_, where *N*_ij_ is the number of observed transitions from state *i* to state *j* and *τ*_*i*_ is the total simulated time for which the system was in ensemble *i*. Here, we only calculated the rate if *N*_*ij*_ > 20. In addition, while rates to/from P/P′ were not considered, for the analysis of kinetics, the system was defined as "not-I1” when a P/P′ configuration was sampled, until the system readopted an I1 configuration. P/P’ configurations were defined as being centered about (*R*_P_, *R*_E_) = (6, 48). Calculated rates are given in Supplementary Table 1.

### Free-energy perturbation calculations

Free-energy perturbation calculations were performed to study the effects of steric-gate modifications (Fig. 5), different A-site tRNA positions (Fig. 6), and electrostatic interactions (Supplementary Fig. 14). In these cases, the change in free energy was calculated according to the relation^68^:

4 $$\Delta F=-{{k}}_{{\rm{B}}}T{\mathrm{ln}}\,{\left\langle \exp \left(-\frac{\Delta U}{{{k}}_{{\rm{B}}}T}\right)\right\rangle }_{0,{\rho }_{1,2}}.$$

Δ*U* is the change in potential energy upon introducing a modification to the model. ${\langle ...\rangle }_{0,{\rho }_{1,2}}$ denotes an ensemble average taken over the simulated frames for which the ribosome adopts a given value of *ρ*_1,2_.

#### Steric-gate perturbations

For the steric-gate calculations (Fig. 5d), Δ*U* was defined as the change in potential energy associated with excluded-volume interactions between tRNA and the gate residues (Fig. 5c).

#### A-site tRNA position perturbations

To assess the influence of the A-site tRNA position on the free energy of the P-site tRNA (Fig. 6c), Δ*U* was defined by a hard-sphere potential between the atoms in the A/P or A/P* structures and the simulated P-site tRNA: Δ*U* = ∞ if the minimal intermolecular distance is <2.5 Å.

#### Electrostatic effects

To obtain an initial insight into the influence of electrostatic interactions, we assigned partial charges to each atom and then applied a screened-electrostatic (Debye–Hückel) potential between the P-site tRNA and the 50S subunit. Charges were based on the amber99sb-ildn forcefield^69^. Since the structure-based model does not explicitly represent hydrogen atoms, the charge on each hydrogen atom in the AMBER forcefield was added to the corresponding heavy atom in the SMOG model. The parameters for the Debye–Hückel potential were taken from ref. ^70^, with a monovalent ion concentration of 100 mM and a uniform dielectric constant of 80. The potential was truncated at 20 Å.

### Reporting summary

Further information on research design is available in the Nature Research Reporting Summary linked to this article.

## Supplementary information

Supplementary Information

Reporting Summary

## Data Availability

All the data are available upon reasonable request.

## References

[1] Schmeing TM, Ramakrishnan V (2009). What recent ribosome structures have revealed about the mechanism of translation. Nature.

[2] Korostelev A, Noller HF (2007). The ribosome in focus: new structures bring new insights. Trends Biochem. Sci..

[3] Frank J, Gao H, Sengupta J, Gao N, Taylor DJ (2007). The process of mRNA-tRNA translocation. Proc. Natl Acad. Sci. USA.

[4] Rodnina MV, Wintermeyer W (2001). Fidelity of aminoacyl-tRNA selection on the ribosome: kinetic and structural mechanisms. Annu. Rev. Biochem..

[5] Frank J, Agrawal R (2000). A ratchet-like inter-subunit reorganization of the ribosome during translocation. Nature.

[6] Valle M (2003). Locking and unlocking of ribosomal motions. Cell.

[7] Moazed D, Noller HF (1989). Intermediate states in the movement of transfer RNA in the ribosome. Nature.

[8] Horan LH, Noller HF (2007). Intersubunit movement is required for ribosomal translocation. Proc. Natl Acad. Sci. USA.

[9] Brilot AF, Korostelev AA, Ermolenko DN, Grigorieff N (2013). Structure of the ribosome with elongation factor G trapped in the pretranslocation state. Proc. Natl Acad. Sci. USA.

[10] Ratje A (2010). Head swivel on the ribosome facilitates translocation by means of intra-subunit tRNA hybrid sites. Nature.

[11] Dunkle JA (2011). Structures of the bacterial ribosome in classical and hybrid states of tRNA binding. Science.

[12] Nguyen K, Whitford PC (2016). Steric interactions lead to collective tilting motion in the ribosome during mRNA–tRNA translocation. Nat. Commun..

[13] Ogle JM, Carter AP, Ramakrishnan V (2003). Insights into the decoding mechanism from recent ribosome structures. Trends Biochem. Sci..

[14] Agirrezabala X (2008). Visualization of the hybrid state of tRNA binding promoted by spontaneous ratcheting of the ribosome. Mol. Cell.

[15] Fischer N, Konevega AL, Wintermeyer W, Rodnina MV, Stark H (2010). Ribosome dynamics and tRNA movement by time-resolved electron cryomicroscopy. Nature.

[16] Frank J (2012). Intermediate states during mRNA–tRNA translocation. Curr. Opin. Struct. Biol..

[17] Korostelev A, Trakhanov S, Laurberg M, Noller HF (2006). Crystal structure of a 70S ribosome-tRNA complex reveals functional interactions and rearrangements. Cell.

[18] Jenner L, Rees B, Yusupov M, Yusupova G (2007). Messenger RNA conformations in the ribosomal E site revealed by X-ray crystallography. EMBO Rep..

[19] Blanchard SC, Kim HD, Gonzalez RL, Puglisi JD, Chu S (2004). tRNA dynamics on the ribosome during translation. Proc. Natl Acad. Sci. USA.

[20] Munro JB (2010). Spontaneous formation of the unlocked state of the ribosome is a multistep process. Proc. Natl Acad. Sci. USA.

[21] Fei J (2009). Allosteric collaboration between elongation factor G and the ribosomal L1 stalk directs tRNA movements during translation. Proc. Natl Acad. Sci. USA.

[22] Cornish PV, Ermolenko DN, Noller HF, Ha T (2008). Spontaneous intersubunit rotation in single ribosomes. Mol. Cell.

[23] Ermolenko DN (2007). Observation of intersubunit movement of the ribosome in solution using FRET. J. Mol. Biol..

[24] Rodnina MV, Savelsbergh A, Wintermeyer W (1999). Dynamics of translation on the ribosome: molecular mechanics of translocation. FEMS Microbiol. Rev..

[25] Rodnina MV, Wintermeyer W (2011). The ribosome as a molecular machine: the mechanism of tRNA–mRNA movement in translocation. Biochem. Soc. Trans..

[26] Pan D, Kirillov SV, Cooperman BS (2007). Kinetically competent intermediates in the translocation step of protein synthesis. Mol. Cell.

[27] Munro JB, Altman RB, O’Connor N, Blanchard SC (2007). Identification of two distinct hybrid state intermediates on the ribosome. Mol. Cell.

[28] Zhou J, Lancaster L, Donohue JP, Noller HF (2014). How the ribosome hands the A-site tRNA to the P site during EF-G–catalyzed translocation. Science.

[29] Bryngelson JD, Wolynes PG (1989). Intermediates and barrier crossing in a random energy-model (with applications to protein folding). J. Phys. Chem.-US.

[30] Bryngelson JD, Onuchic JN, Socci ND, Wolynes PG (1995). Funnels, pathways, and the energy landscape of protein-folding—a synthesis. Proteins.

[31] Onuchic J, Luthey-Schulten Z, Wolynes P (1997). Theory of protein folding: the energy landscape perspective. Annu. Rev. Phys. Chem..

[32] Kubelka J, Hofrichter J, Eaton WA (2004). The protein folding ‘speed limit’. Curr. Opin. Struct. Biol..

[33] Thirumalai D, Hyeon C (2005). RNA and protein folding: common themes and variations. Biochemistry.

[34] Kim H (2014). Protein-guided RNA dynamics during early ribosome assembly. Nature.

[35] Pierro MD, Potoyan DA, Wolynes PG, Onuchic JN (2018). Anomalous diffusion, spatial coherence, and viscoelasticity from the energy landscape of human chromosomes. Proc. Natl Acad. Sci. USA.

[36] Zhuravlev PI, Papoian GA (2009). Molecular noise of capping protein binding induces macroscopic instability in filopodial dynamics. Proc. Natl Acad. Sci. USA.

[37] Dashti A (2014). Trajectories of the ribosome as a Brownian nanomachine. Proc. Natl Acad. Sci. USA.

[38] Levi M, Noel JK, Whitford PC (2019). Studying ribosome dynamics with simplified models. Methods.

[39] Bock LV, Blau C, Vaiana AC, Grubmüller H (2015). Dynamic contact network between ribosomal subunits enables rapid large-scale rotation during spontaneous translocation. Nucleic Acid Res..

[40] Adamczyk, A. J. & Warshel, A. Converting structural information into an allosteric-energy-based picture for elongation factor tu activation by the ribosome. *Proc. Natl. Acad. Sci. USA* **108**, 9827–9832 (2011). 10.1073/pnas.1105714108 PMC3116401 21617092

[41] Trobro S, Aqvist J (2008). Role of ribosomal protein L27 in peptidyl transfer. Biochemistry.

[42] Whitford PC (2009). An all-atom structure-based potential for proteins: bridging minimal models with all-atom empirical forcefields. Prot. Struct. Funct. Bioinform..

[43] Noel JK (2016). SMOG 2: A versatile software package for generating structure-based models. PLoS Comput. Biol..

[44] Bock LV (2013). Energy barriers and driving forces in tRNA translocation through the ribosome. Nat. Struct. Mol. Biol..

[45] Whitford PC, Sanbonmatsu KY (2013). Simulating movement of tRNA through the ribosome during hybrid-state formation. J. Chem. Phys..

[46] Hyeon C, Thirumalai D (2011). Capturing the essence of folding and functions of biomolecules using coarse-grained models. Nat. Commun..

[47] Chan, H. S., Zhang, Z., Wallin, S. & Liu, Z. Cooperativity, local–nonlocal coupling, and nonnative interactions: principles of protein folding from coarse-grained models. *Annu. Rev. Phys. Chem*. **62**, 301–326 (2011). 10.1146/annurev-physchem-032210-103405 21453060

[48] Okazaki K, Takada S (2008). Dynamic energy landscape view of coupled binding and protein conformational change: induced-fit versus population-shift mechanisms. Proc. Natl Acad. Sci. USA.

[49] Nguyen K, Yang H, Whitford PC (2017). How the ribosomal A-site finger can lead to tRNA species-dependent dynamics. J. Phys. Chem. B.

[50] Fei J, Kosuri P, Macdougall D, Gonzalezjr R (2008). Coupling of ribosomal L1 stalk and tRNA dynamics during translation elongation. Mol. Cell.

[51] Kim HD, Puglisi JD, Chu S (2007). Fluctuations of transfer RNAs between classical and hybrid states. Biophys. J..

[52] Graf M (2018). Visualization of translation termination intermediates trapped by the Apidaecin 137 peptide during RF3-mediated recycling of RF1. Nat. Commun..

[53] Svidritskiy E, Demo G, Loveland AB, Xu C, Korostelev AA (2019). Extensive ribosome and RF2 rearrangements during translation termination. eLife.

[54] Noel JK, Chahine J, Leite VBP, Whitford PC (2014). Capturing transition paths and transition states for conformational rearrangements in the ribosome. Biophys. J..

[55] Levi M, Whitford PC (2019). Dissecting the energetics of subunit rotation in the ribosome. J. Phys. Chem. B.

[56] Best RB, Hummer G (2005). Reaction coordinates and rates from transition paths. Proc. Natl Acad. Sci. USA.

[57] Hummer G (2004). From transition paths to transition states and rate coefficients. J. Chem. Phys..

[58] Whitford PC, Blanchard SC, Cate JHD, Sanbonmatsu KY (2013). Connecting the kinetics and energy landscape of tRNA translocation on the ribosome. PLoS Comput. Biol..

[59] Noel JK, Whitford PC (2016). How EF-Tu can contribute to efficient proofreading of aa-tRNA by the ribosome. Nat. Commun..

[60] Yang H, Noel JK, Whitford PC (2017). Anisotropic fluctuations in the ribosome determine tRNA kinetics. J. Phys. Chem. B.

[61] Dhar A (2010). Structure, function, and folding of phosphoglycerate kinase are strongly perturbed by macromolecular crowding. Proc. Natl Acad. Sci. USA.

[62] Bernier CR, Petrov AS, Kovacs NA, Penev PI, Williams LD (2018). Translation: the universal structural core of life. Mol. Biol. Evol..

[63] Jenner LB, Demeshkina N, Yusupova G, Yusupov M (2010). Structural aspects of messenger RNA reading frame maintenance by the ribosome. Nat. Struct. Mol. Biol..

[64] Noel JK, Whitford PC, Onuchic JN (2012). The shadow map: a general contact definition for capturing the dynamics of biomolecular folding and function. J. Phys. Chem. B.

[65] Lindahl E, Hess B, van der Spoel D (2001). Gromacs 3.0: a package for molecular simulation and trajectory analysis. J. Mol. Mod..

[66] Hess B, Kutzner C, van der Spoel D, Lindahl E (2008). GROMACS 4: algorithms for highly efficient, load-balanced, and scalable molecular simulation. J. Chem. Theory Comput..

[67] Yang, H. et al. Diffusion of tRNA inside the ribosome is position-dependent. *J. Chem. Phys*. **151**, 085102 (2019). 10.1063/1.5113814 31470725

[68] Zwanzig RW (1954). High-temperature equation of state by a perturbation method. I. nonpolar gases. J. Chem. Phys..

[69] Lindorff-Larsen K (2010). Improved side-chain torsion potentials for the Amber ff99SB protein force field. Prot. Struct. Funct. Bioinform..

[70] Givaty O, Levy Y (2009). Protein sliding along DNA: dynamics and structural characterization. J. Mol. Biol..

