# Supplementary material for: A steric gate controls P/E hybrid-state formation of tRNA on the ribosome
Source: Nat Commun. 2020 Nov 11;11:5706. doi: 10.1038/s41467-020-19450-0 (PMC7658246; doi:10.1038/s41467-020-19450-0)
Supplement: Supplementary file 1 — Supplementary Information [file 41467_2020_19450_MOESM1_ESM.pdf]

Supplementary Information for "A steric gate controls P/E hybrid-state formation of tRNA on the ribosome"

Mariana Levi,[1] Kelsey Walak,[1] Ailun Wang,[2] Udayan Mohanty,[2] and Paul C. Whitford[1,3]

[1]*Department of Physics, Northeastern University, Dana Research Center 123, 360 Huntington Ave, Boston, MA 02115*

[2]*Department of Chemistry, Boston College*

[3]*Center for Theoretical Biological Physics, Northeastern University, 360 Huntington Ave, Boston, MA 02115*

(Dated: October 15, 2020)

SUPPLEMENTARY NOTE 1

We use the following nomenclature to describe various conformations of tRNA (predicted, or experimentally-reported) on the ribosome.

- A/A-P/P - Classical pre-translocation configuration. One tRNA is bound to the A sites of the small and large subunit, while the second tRNA is bound to the P sites. The small subunit body and head are in unrotated positions. In the current study, calculations involving A/A-P/P use PDB entry 4V6F [1]. Since this structure contains tRNA molecules in the A, P and E sites, the E-site tRNA was removed to define the pre-translocation configuration. The A/A-P/P configuration is shown in Fig. 1.

- P/P-E/E - Classical post-translocation configuration. One tRNA is bound to the P sites of the small and large subunit, while the second tRNA is bound to the E sites. The small subunit body and head are in unrotated positions. For our calculations, we use PDB entry 4V6F [1]. Since this structure contains tRNAs in the A, P and E sites, the A-site tRNA was removed to define the post-translocation configuration. The P/P-E/E structure is shown in Supplementary Fig. 1.

- $\mathrm{TI}^{\mathrm{Pre}}$ - Consistent with the nomenclature of Ratje et al. [2], this refers to a configuration in which a tRNA molecule is in a P/E configuration, while the small subunit body is rotated $\sim 7°$, relative to the classical orientation. The cryo-EM reconstructions reported in Ref. [2] lacked an A-site tRNA molecule. Accordingly, subsequent structural models were constructed that included an A-site tRNA molecule, where biochemical arguments were used to approximate its position [3]. Since the current simulations aimed to explore the influence of the A-site tRNA during P/E formation, the latter models were used for all calculations. However, with the exception of the A-site tRNA, there are no significant differences between the two structural models.

- P/P' - This notation denotes a small ensemble of configurations that is sampled in the presented simulations. The tRNA interacts with the P site of both subunits. In contrast to the classical P/P configuration, the small subunit is rotated by $\sim 7°$. This ensemble is heavily disfavored and is only transiently sampled. Further, as described in the main text, this configuration is not accessible once the A-site tRNA has moved towards the

P site (i.e. A/P or A/P* configurations, defined below). Specifically, when the A-site tRNA binds G2251 in the P site, it is not possible for the P-site tRNA to also approach G2251, which thereby restricts the molecule to larger values of R_{P}. A representative P/P' configuration is shown in Fig. 2c.

- I1 - Intermediate 1 is predicted by the current model. It is associated with a P-site tRNA that is bound to the P site of the small subunit, while the elbow and 3'-CCA end are displaced slightly from the P site on the large subunit. The small subunit is rotated by $\sim 7°$. A representative I1 configuration is shown in Figs. 2d, 4 and 6d.

- I2 - Intermediate 2 is predicted by the current model. It is associated with a P-site tRNA that is bound to the P site of the small subunit, while the elbow and 3'-CCA end are near the E site of the large subunit. The small subunit is rotated by $\sim 7°$. An I2 configuration is shown in Figs. 2e and 4d.

- P/E - This configuration has been reported experimentally, and it is captured by the current model. The P-site tRNA is bound to the P site of the small subunit and E site of the large subunit. The small subunit is rotated by $\sim 7°$. A P/E configuration is shown in Figs. 1b and 2f.

- NE - The "Near E site" configuration is sampled in the presented simulations. The P-site tRNA is bound to the P site of the small subunit, while the 3'-CCA end appears to have passed the E site of the large subunit. The small subunit is rotated by $\sim 7°$. A NE configuration is shown in Supplementary Fig. 10.

- A/P - Consistent with the notation of Brilot et al. [4], this refers to a tRNA molecule that is bound to the A site of the small subunit, while the tRNA elbow is near the A site of the large subunit and the 3'-CCA end is displaced to the P site of the large subunit. The small subunit is rotated by $\sim 7°$. This orientation of the A-site tRNA is similar the "A/P*" configuration described in Ref. [3]. The A/P configuration is shown in Figs. 6a and 6b.

- A/P* - Consistent with the notation of Brilot et al. [4], this refers to a tRNA molecule that is bound to the A site of the small subunit, while the tRNA elbow and 3'-CCA end are displaced to the P site of the large subunit. The small subunit is rotated by $\sim 7°$.

This orientation of the A-site tRNA is similar the "A/P" configuration described in Ref. [3]. The A/P* configuration is shown in Fig. 6b.

- Structure III - This refers to hybrid-like configurations that have been identified in cryo-EM studies of the ribosome with release factors bound [5, 6]. The tRNA position is similar to the predicted I1 intermediate (Supplementary Fig. 11). The small subunit is rotated by $\sim 5^{\circ}$.

SUPPLEMENTARY METHODS

Sequence analysis of protein L33

The following steps were performed to assess the level of conservation around position 29 in L33. Bacterial L33 sequences were obtained from the Uniprot database [7] (reviewed sequences, only) using the search

```
"ribosomal protein l33" taxonomy:bacteria AND reviewed:yes
```

This yielded 842 sequences, two of which were non-L33 sequences. The remaining 840 sequences were aligned using the ClustalW algorithm [8], as implemented in the MultiSeq module [9] of VMD [10]. All references to residue positions denote the corresponding position in Thermus Thermophilus.

SUPPLEMENTARY FIGURES

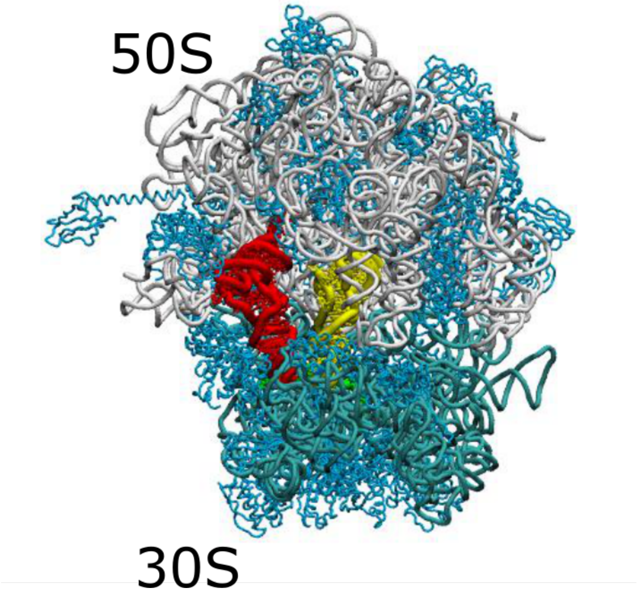

SUPPLEMENTARY FIG. 1. **Classical P/P-E/E conformation.** Upon completion of tRNA translocation, the tRNA molecule that was initially in the A site is displaced to the P site, and it adopts the P/P configuration (yellow). Similarly, the initially-P/P tRNA reaches the E/E configuration (red). After translocation, the subunits return to their "classical" unrotated orientation. PDB ID 4V6F [1] is shown. All structural representations were generated with VMD [10].

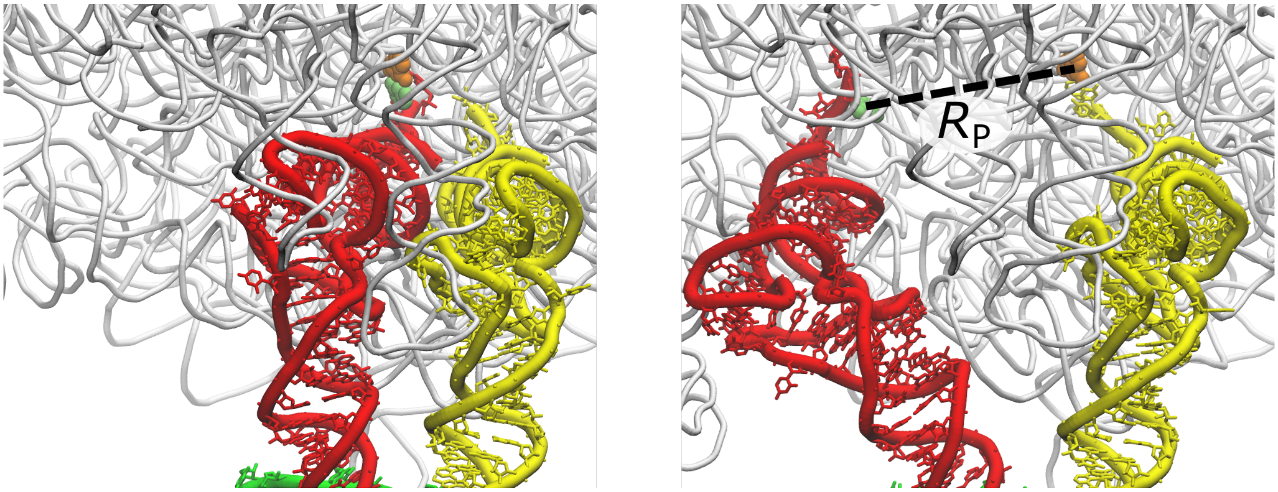

SUPPLEMENTARY FIG. 2. **Defining R_P.** The position of the 3'-CCA end from the P site of the large subunit is described by R_P: distance between the geometric centers of the functional groups of C75 in the tRNA (shown in light green spheres) and G2251 of the 23S (orange spheres). Structures of P/P [1] and P/E ($\mathrm{TI}^{\mathrm{Pre}}$) [3] configurations are shown.

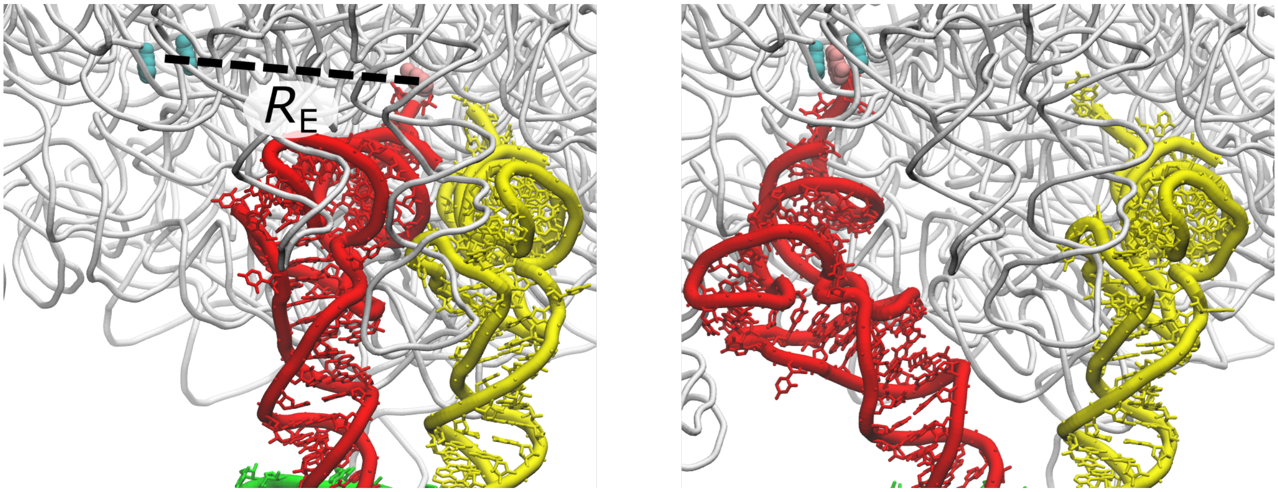

SUPPLEMENTARY FIG. 3. **Defining R_E.** The position of the 3'-CCA end from the E site of the large subunit is described by R_E: distance between the geometric centers of the functional groups of A76 in the tRNA (shown in light pink spheres) and G2421/A2422 of the 23S (cyan spheres). Same structures are shown as in Supplementary Fig. 2.

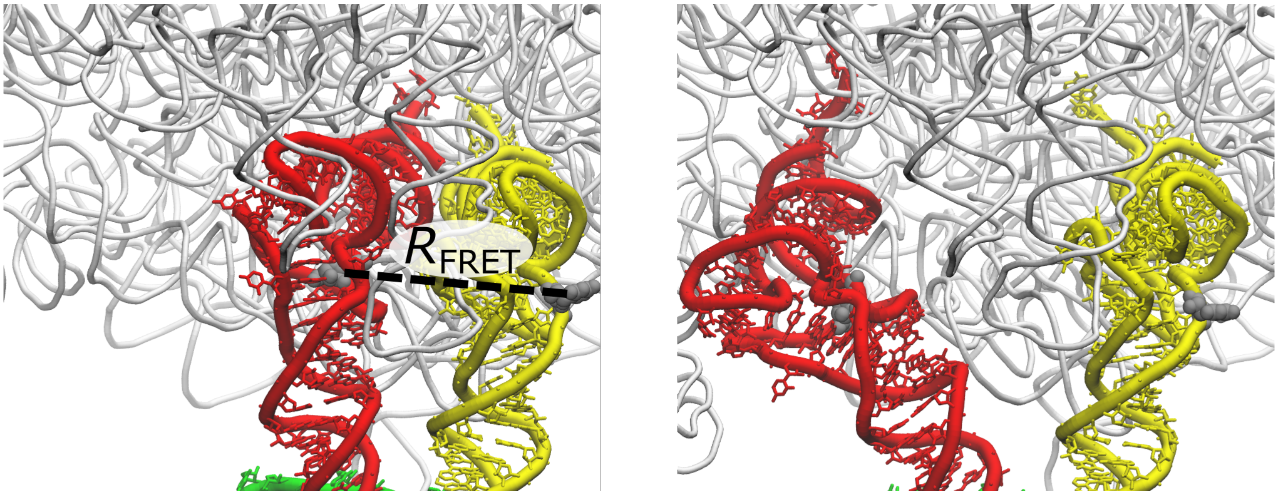

SUPPLEMENTARY FIG. 4. **Defining R_{FRET}.** The position of the tRNA elbow is described by R_{FRET}: distance between the geometric centers of U47 (gray spheres) in the A-site tRNA (yellow) and U8 (gray spheres) in the P-site tRNA (red). These residues were used to compare with single-molecule experiments, which typically label the U47-U8 pair. Same structures are shown as in Supplementary Fig. 2.

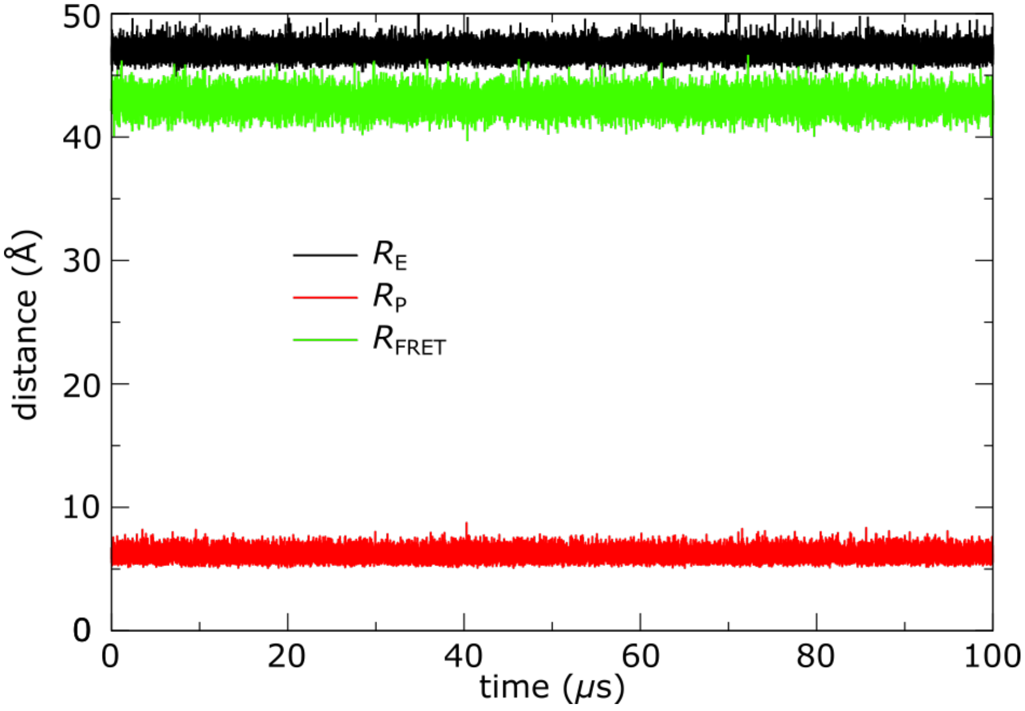

SUPPLEMENTARY FIG. 5. **Dynamics of tRNA when the ribosome is unrotated.** In this simulation, the unrotated configuration was defined as the potential energy minimum. When no position restraints were imposed on the system, the system adopts rotation angles of $\sim 0°$ (angle defined in [11]). Consistent with biochemical and single-molecule inferences, when the subunits are unrotated, the tRNA molecule remains in the P/P configuration, where $R_{\mathrm{P}} \approx 6$Å (red), $R_{\mathrm{FRET}} \approx 43$Å (green) and $R_{\mathrm{E}} \approx 46$Å (black).

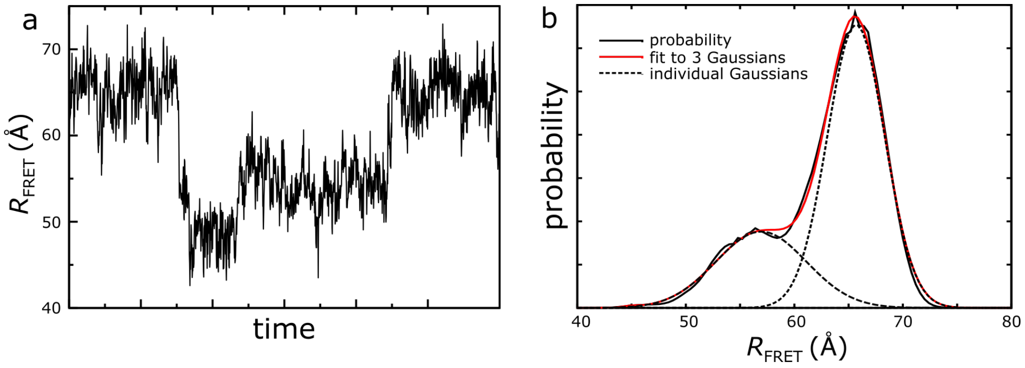

SUPPLEMENTARY FIG. 6. **Dynamics of tRNA for a rotated ribosome.** When the small subunit is restrained to a rotated orientation (TI^{pre} configuration of Refs. [2, 3]), there are multiple distinct ensembles that are accessible to the tRNA molecule. **a** Representative time trace that illustrates the overall dynamics along $R_{\rm FRET}$. **b** Since there appeared to be three distinct states in the time traces, the probability distribution as a function of $R_{\rm FRET}$ was fit to the sum of three Gaussians: $\Sigma_{i=1}^{3} A_{\rm i} \exp^{-B_{\rm i}(R_{\rm FRET}-r_{\rm i})^2}$. The fitted parameters are: $(A_{\rm i}, B_{\rm i} * \mathring{\rm A}^2, r_{\rm i}/\mathring{\rm A})$= (1,0.943,45.0), (61.3,0.0287,56.9), (227,0.0713,65.7). While a low-$R_{\rm FRET}$ ensemble ($r_{\rm i}$ = 45Å) is visible in time traces, its amplitude is negligible, relative to the other two peaks. This is consistent with P/P-like configurations being heavily disfavored when the ribosomal subunits adopt a rotated orientation.

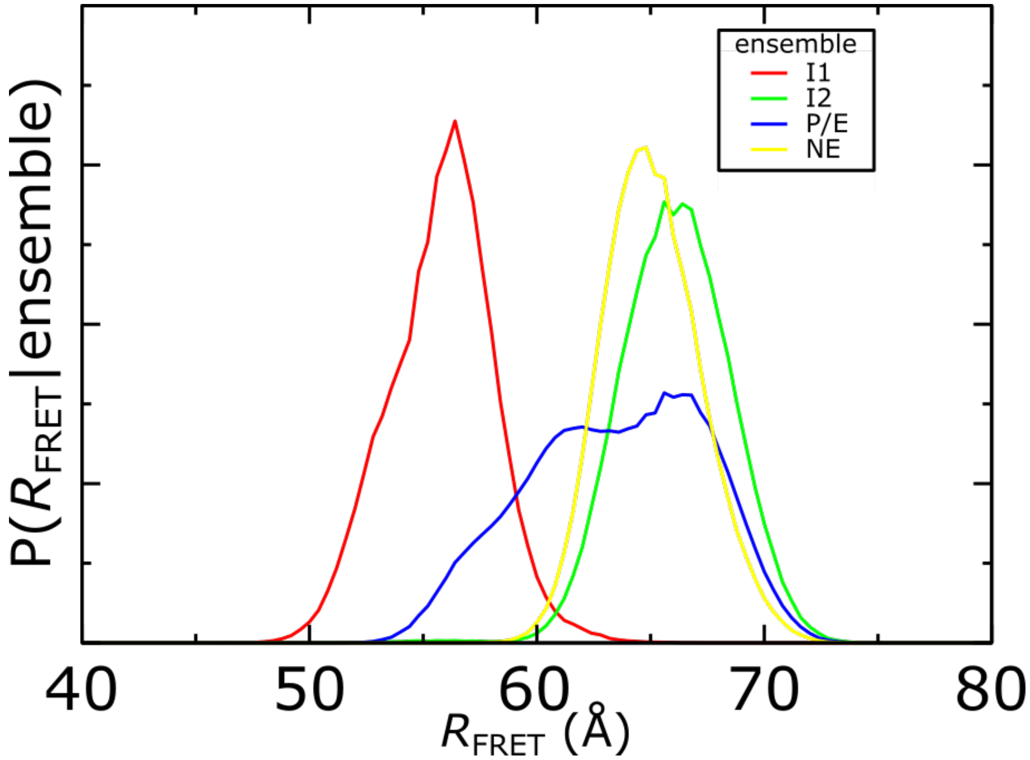

SUPPLEMENTARY FIG. 7. **Distribution of R_{FRET} values for different ensembles.** To address how the states identified with R_{P} and R_{E} can be related to single-molecule FRET measurements, we calculated the distribution of R_{FRET} values for each ensemble. The definition of each state is based on R_{P}-R_{E} values, as described under *Calculating microscopic rates* in the main text (See Supplementary Fig. 12). When the ribosome is unrotated and the 3'-CCA end is in the P site (P/P ensemble), the tRNA elbows maintain low values of R_{FRET} ($\sim$ 43Å; Supplementary Fig. 5). Relative to the P/P ensemble, the I1 ensemble (red) is associated with a $\sim$ 10Å displacement of the tRNA elbow. The I2 (green), P/E (blue) and NE (yellow) ensembles exhibit distributions along R_{FRET} that are peaked around 65Å. Note that, since each distribution is normalized, the relative heights should not be interpreted.

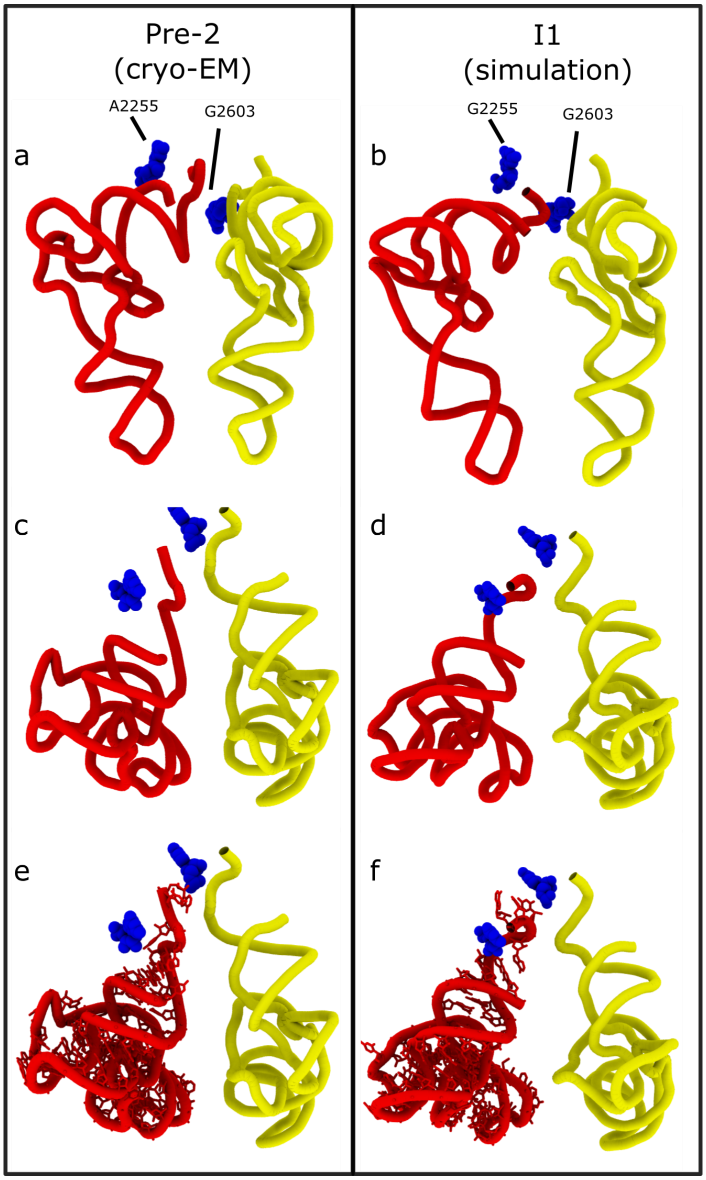

SUPPLEMENTARY FIG. 8. **Comparison of the Pre-2 configuration in cryo-EM with the predicted I1 ensemble.** Structural model of the Pre-2 conformation (PDB: 4V71) described by Bock et al. [12] (panels **a**, **c**, **e**), which was built using flexible fitting methods with a cryo-EM density (EMD-1717) reported by Fischer et al. [13]. Representative snapshot of the I1 ensemble (panels **b**, **d**, **f**), obtained in the present simulations. **a,b** Despite differences in molecular composition (tRNA^{fMet} and tRNA^{Val} in cryo-EM, tRNA^{Phe} in simulations) and conditions (non-equilibrium reverse translocation conditions in experiments, equilibrium P/E formation conditions in simulations), the overall positions of the tRNA molecules are similar for Pre-2 and I1 configurations. In both cases, the P-site tRNA elbow is displaced away from the A-site tRNA, relative to the classical P/P orientation (Supplementary Fig. 2, left panel), while the 3'-CCA end remains near the P site. To highlight the similar positioning of the 3'-CCA end, rRNA residues A2255 (G2255 in TT) and G2603 are shown (blue spheres). **c,d** Top views of panels A and B. In both systems, the 3'-CCA end is found to be in an extended, non-base-stacked configuration (panels **e, f**).

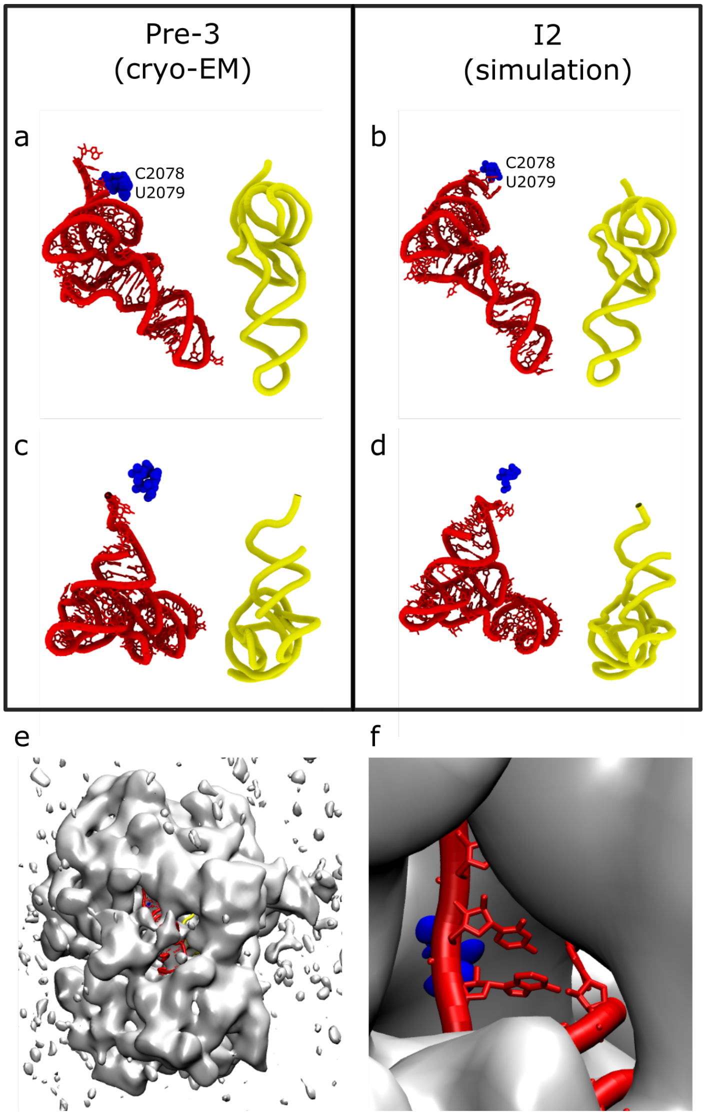

SUPPLEMENTARY FIG. 9. **Comparison of the Pre-3 configuration in cryo-EM with the predicted I2 ensemble.** Structural model of the Pre-3 conformation (PDB: 4V70) described by Bock et al. [12] (panels **a, c**), which is based on the cryo-EM density (EMD-1718) of Fischer et al. [13]. Representative snapshot of the I2 ensemble (panels **b, d**), obtained in the present simulations. **a,b** Both models are associated with the tRNA elbow adopting a P/E-like position. **c,d** The most notable difference between the structural models is that the 3'-CCA tail of Pre-3 model is more extended, leading to a position that is "above" (from the perspective shown) rRNA residues C2078/U2079, whereas the I2 ensemble can access configurations in which the tail is "below." **e** Unfiltered cryo-EM reconstruction of Fischer et al., shown with the Pre-3 model of Bock et al. The majority of the Pre-3 tRNA molecule is well within the high-density region, though the 3'-CCA tail yields a minimal density in this state (panel **f**). This implies there is a significant degree of conformational heterogeneity in the tail, where it may sample a range of configurations in the vicinity of C2078/U2079.

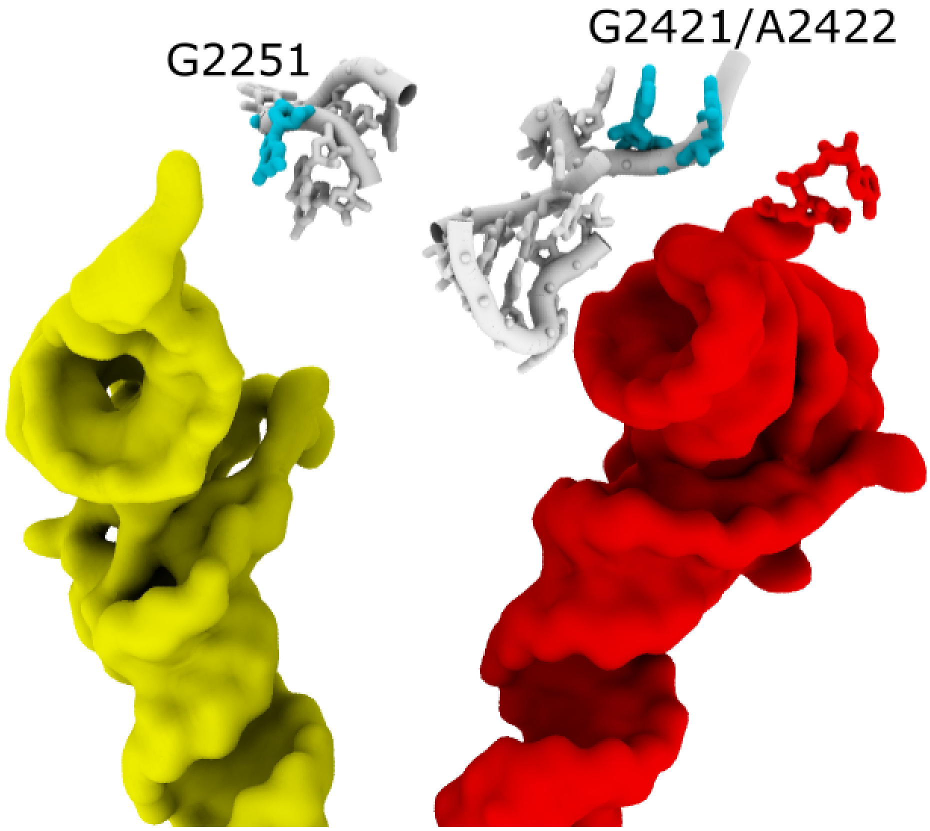

SUPPLEMENTARY FIG. 10. **Representative snapshot of the NE ensemble.** In addition to sampling the P/P', I1, I2 and P/E ensembles, there is another distinct set of configurations sampled ("near E site", NE). These configurations correspond to a tRNA elbow position that is similar to the P/E ensemble, though the 3'-CCA end is "flipped," where it adopts positions that are distal to both the P site (G2251) and E site (G2421/A2422). As described in the main text, we find this ensemble is off-route during P/E formation, and it will not be further analyzed. In support of this non-dominant/off-route assignment, this ensemble has not been reported in experiments. In the current study, we show that this ensemble is sterically accessible, though a more complete energetic model could find this ensemble to be disfavored. Interestingly, the cryo-EM study of Fischer et al. [13] did report a state (Pre-3) in which the tRNA elbow is positioned similarly, while a low density was obtained for the 3'-CCA end (Supplementary Fig. 9f). Accordingly, an alternate possibility is that the previously reported Pre-3 state represents an average that includes some NE-like configurations.

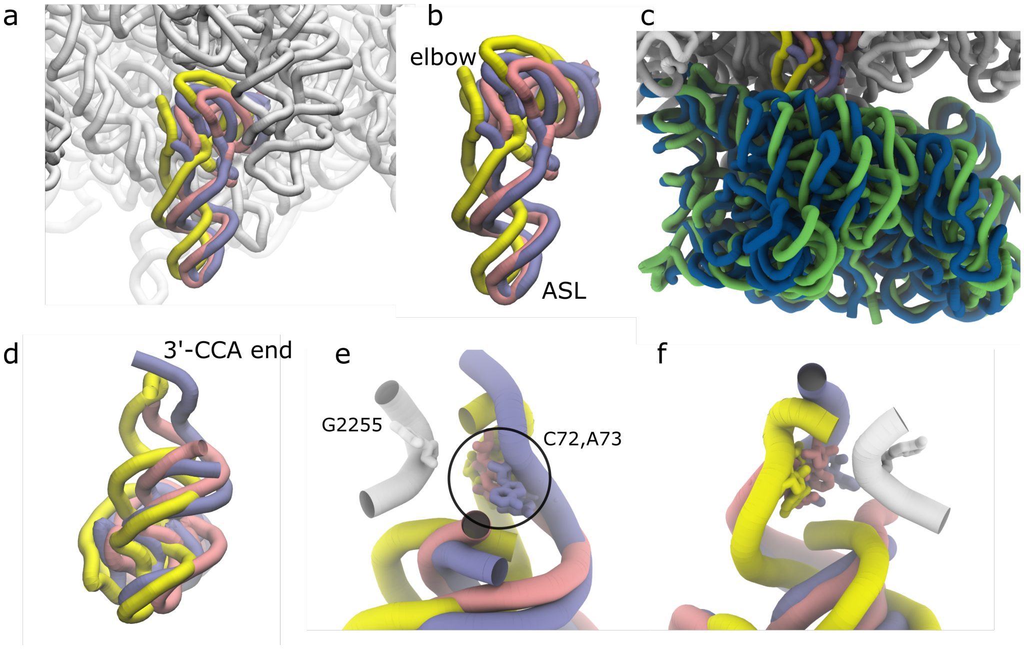

SUPPLEMENTARY FIG. 11. **I1 intermediate is similar to Structure III of tRNA when release factors bind the ribosome.** **a** Representative snapshot of the I1 ensemble (yellow), shown with cryo-EM structures obtained with release factors (RF) bound (PDB 6OGF [5], pink; 6GXN [6], purple). tRNA molecules are shown, after alignment of the 23S rRNA (white). The overall positions of the tRNA molecules are similar, though there are $5-10$Å displacements of I1 tRNA atoms, relative to the RF configuration. **b** Same as panel **a**, without the 23S rRNA shown. **c** Differences in the tRNA position may be attributed to the differential degree of small subunit body and head rotation exhibited in the experimental structures ($\sim 5°$ and $\sim 1°$; PDB 6GXN shown in green) and the simulation ($\sim 7°$ and $\sim 4°$; blue). **d** Same as panel **b**, rotated $\sim 90°$ about the horizontal axis. **e** Close-up view of the experimental and simulated 3'-CCA tails. rRNA shown in white, with G2255 shown with sticks. C72 and A73 of each tRNA structure shown in stick representation. As a note, A73 was the terminal residue modeled in PDB 6OGF, consistent with a disordered 3'-CCA tail. For all three models, C72 and A73 are near G2255, suggesting a common steric obstacle may favor this position. **f** Rotated view ($\sim 180°$ about vertical axis) of panel **e**.

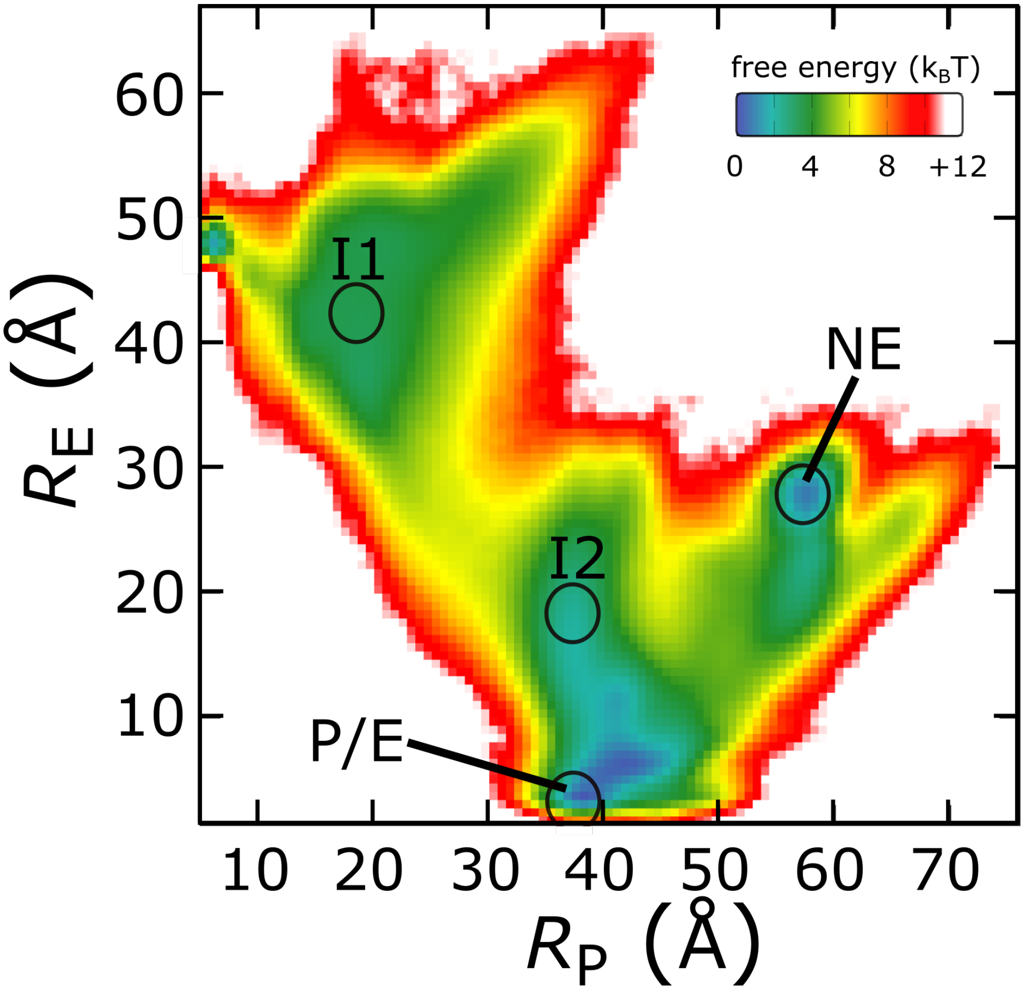

SUPPLEMENTARY FIG. 12. **Definitions of conformational states/ensembles** For the analysis of individual ensembles and the rates of interconversion, each state was defined by a range of values in $R_{\mathrm{P}} - R_{\mathrm{E}}$-space (ovals). See *Calculating microscopic rates* for definitions.

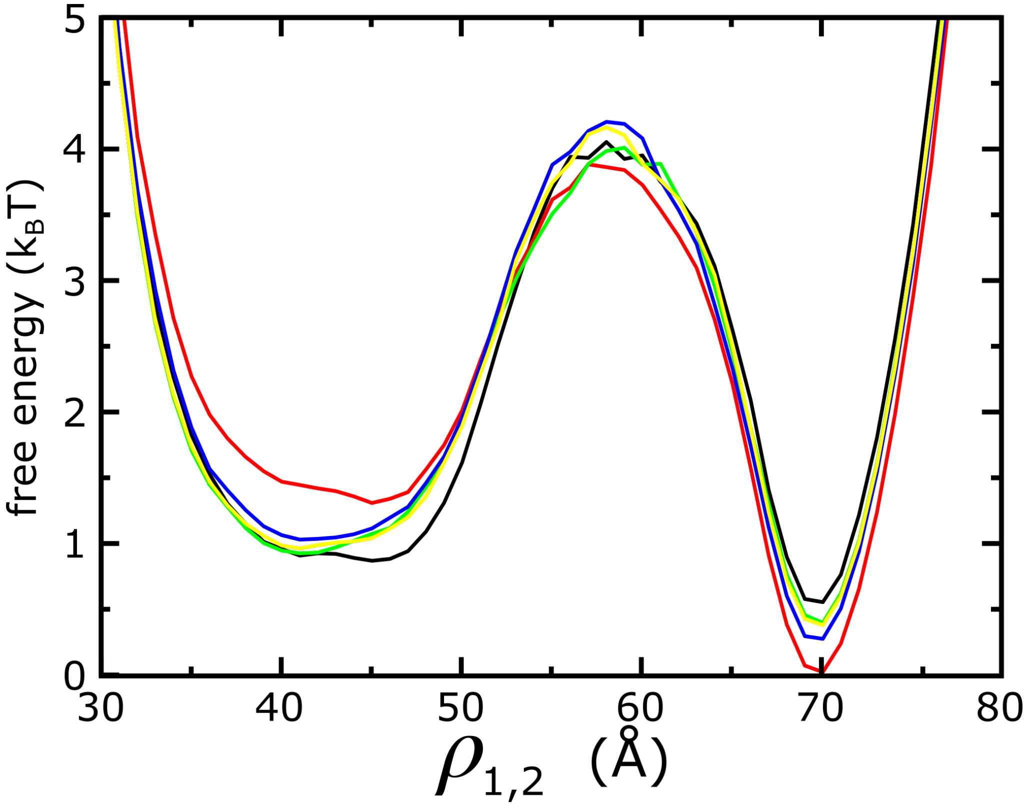

SUPPLEMENTARY FIG. 13. **Free-energy barrier height does not depend on simulated time.** The full data set included 10 simulations of comparable duration. To verify that the calculated barrier was robust to the sampling, we recalculated the free energy using 2 simulations, each. The calculated barrier height was robust across the 5 sets of runs. Each color represents a different subset of the data.

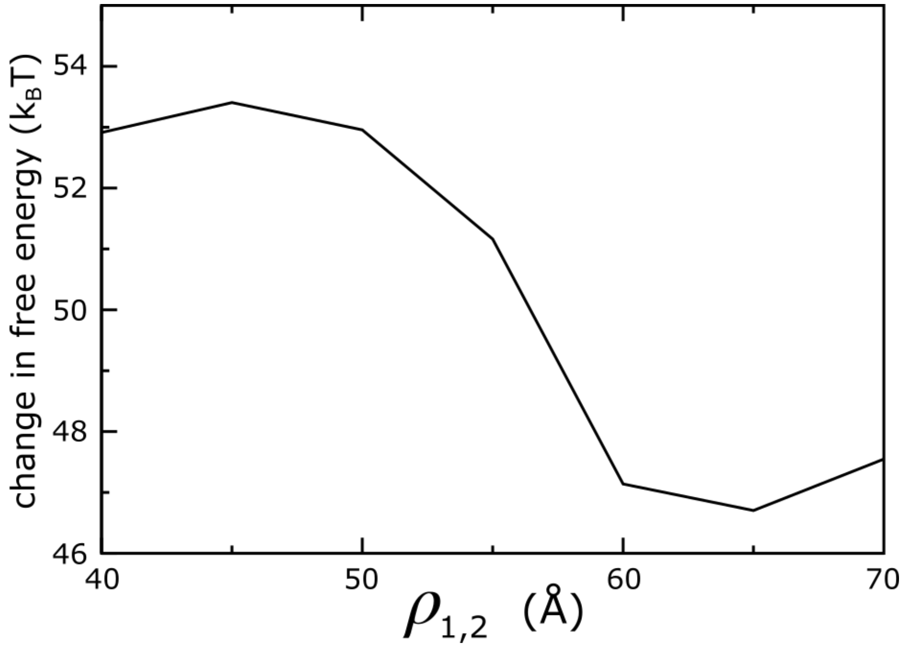

SUPPLEMENTARY FIG. 14. **Electrostatic repulsion does not amplify steric barrier.** Change in free energy upon introduction of screened Debye-Hückel interactions between tRNA and the ribosome. Due to the net negative charge of RNA, backbone-backbone interactions are repulsive when using a DH potential. Accordingly, the nearly monotonically-decreasing change in free-energy reflects the generally less-dense backbone interactions in the TSE and I2 ensembles, relative to I1.

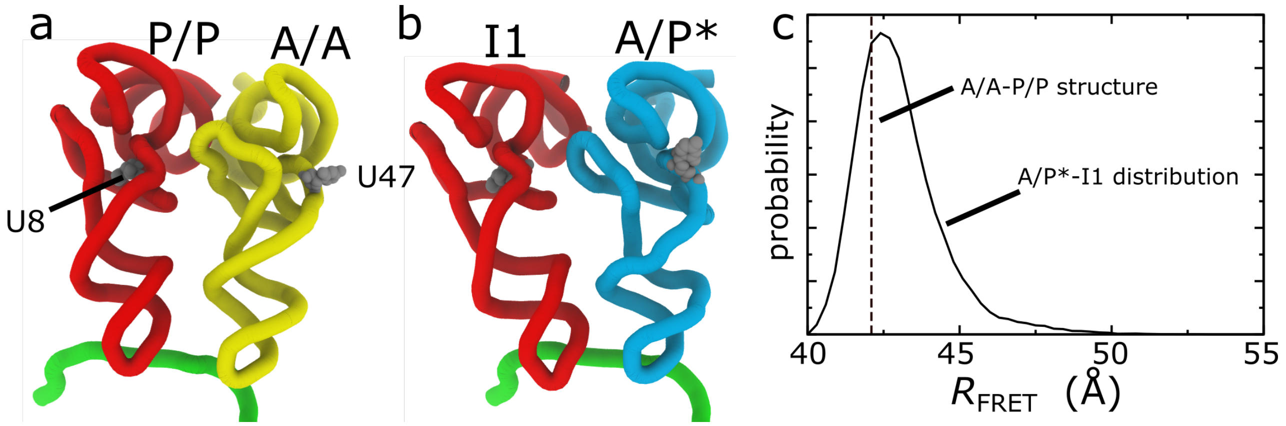

SUPPLEMENTARY FIG. 15. **Multiple configurations may explain high-FRET states.** **a** Structure of the A/A-P/P configuration (PDB entry 4V6F [1]). **b** Representative snapshot of an I1 configuration, with an A/P*-configured tRNA (PDB: 4V7D [4]) aligned to the simulated system (see main text for description). **c** Distribution of $R_{\rm FRET}$ values for the I1 ensemble, with the distances calculated between the simulated P-site tRNA and the A/P* cryo-EM structure. The peak of the distribution is near ($<$ 1Å difference) the value found in the A/A-P/P configuration ($R_{\rm FRET} \sim 42$Å), indicating that high-FRET signals are compatible with either combination of tRNA positions.

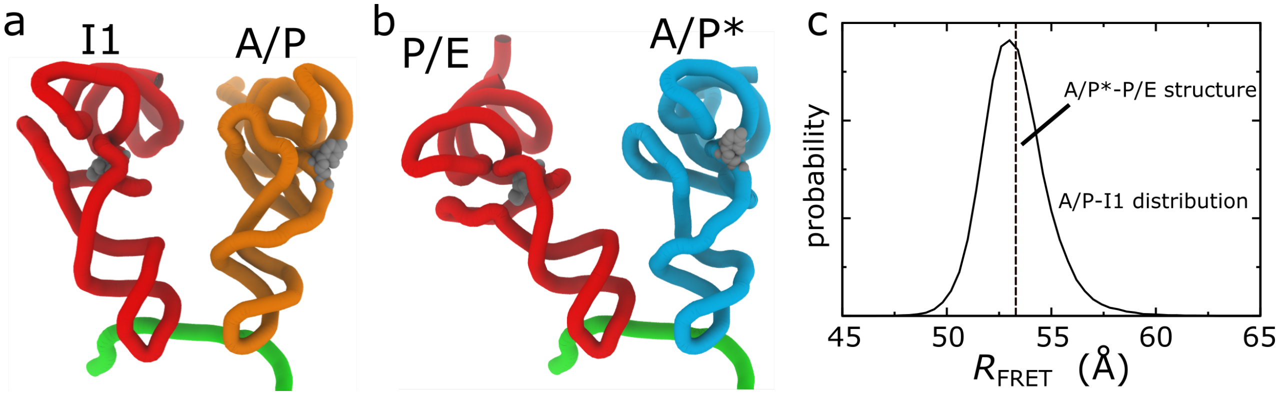

SUPPLEMENTARY FIG. 16. **Multiple configurations may explain mid-FRET states.** **a** Simulated snapshot of an I1-configured tRNA with a cryo-EM structure of an A/P configuration (PDB: 4V7C [4]) aligned. **b** Cryo-EM structure of the A/P*-P/E configuration (PDB: 4V6D [4]). **c** Distribution of R_{FRET} values for the simulated I1 ensemble, where the distances were calculated between the simulated P-site tRNA and the cryo-EM A/P structure. The value obtained from the A/P*-P/E configuration is near ($<$ 1Å difference) the peak of the A/P-I1 distribution ($R_{\mathrm{FRET}} \sim$ 53Å), indicating that mid-FRET signals are compatible with either combination of tRNA positions.

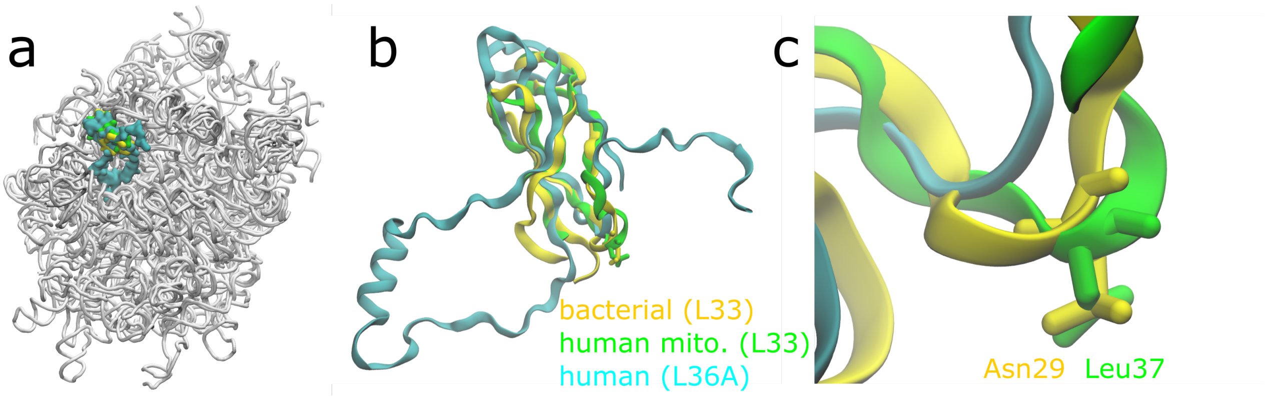

SUPPLEMENTARY FIG. 17. **Comparison of bacterial, human and human mitochondrial ribosomes.** **a** Recent experimental structures of the large subunit rRNA in bacteria (PDB: 6N1D [14]), human (PDB: 6Y0G [15]) and human mitochondria (PDB: 6I9R). rRNA for all three is shown in white, after structure alignment. Alignment was performed based on the large subunit rRNA, using the STAMP algorithm [16], as implemented in VMD [10]. Bacterial and human mitochondrial protein L33 are shown in yellow and green. Human ribosomal protein L36A is shown in cyan. **b** Close-up view of panel A, with proteins shown in ribbon representation. While bacterial and mitochondrial structures of L33 overlap well, L36A is a structural circular permutant. That is, in L36A, the residues that overlap with the termini of L33 are connected by an extended linker. **c** Residue-level view of Asn29 in bacteria. While the mitochondrial structure overlaps with bacteria, the composition is distinct, where Asn29 in bacteria overlaps with Leu37 in mitochondria. The corresponding residues in human do not overlap well in this region. STAMP alignment of L36A with a circularly-permuted sequence (residues 60-106, then 2-59, with the 29-59 insertion removed) did not improve the alignment around Asn29 (not shown).

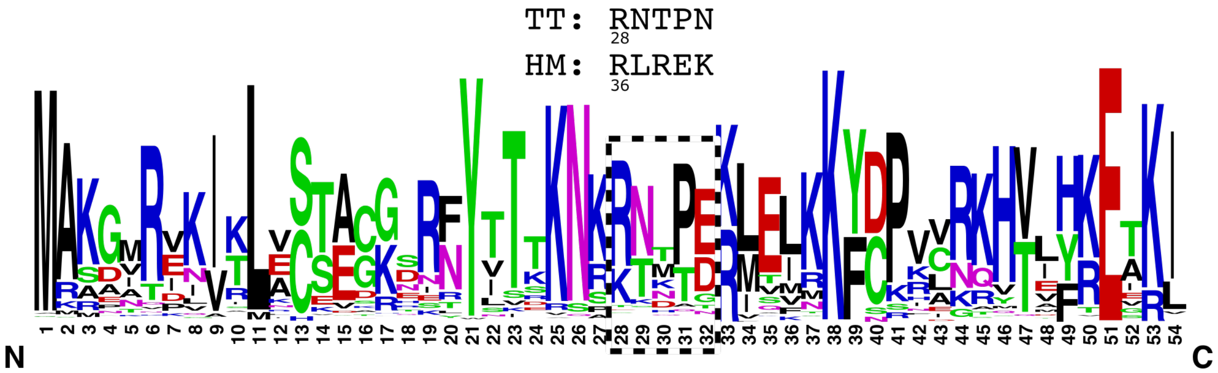

SUPPLEMENTARY FIG. 18. **Sequence conservation of bacterial protein L33.** Top) Sequence of T. Thermophilus (TT) and human mitochondrial (HM) protein L33, beginning with R28 and R36. While R28 in TT was found to structurally align with R36 in HM (Supplementary Fig. 17), the downstream sequences differ. Bottom) Sequence conservation in bacterial L33 proteins. Aligned numbering corresponds to TT. The height of each stack of letters is proportional to negative of the sequence entropy at each position. Within a stack, the height of each letter represents the relative frequency of the corresponding residue at that position. As discussed in the main text, for residues 28-32, there is typically a small number of most-common residues present at each position. Further, as is apparent in the figure, the residues found in positions 37-40 of the human mitochondrial L33 are rarely found in the corresponding positions of bacteria. Letters are colored by residue type (green, polar; blue, basic; red, acidic; black, hydrophobic). Sequence logo image prepared with the WebLogo server [17].

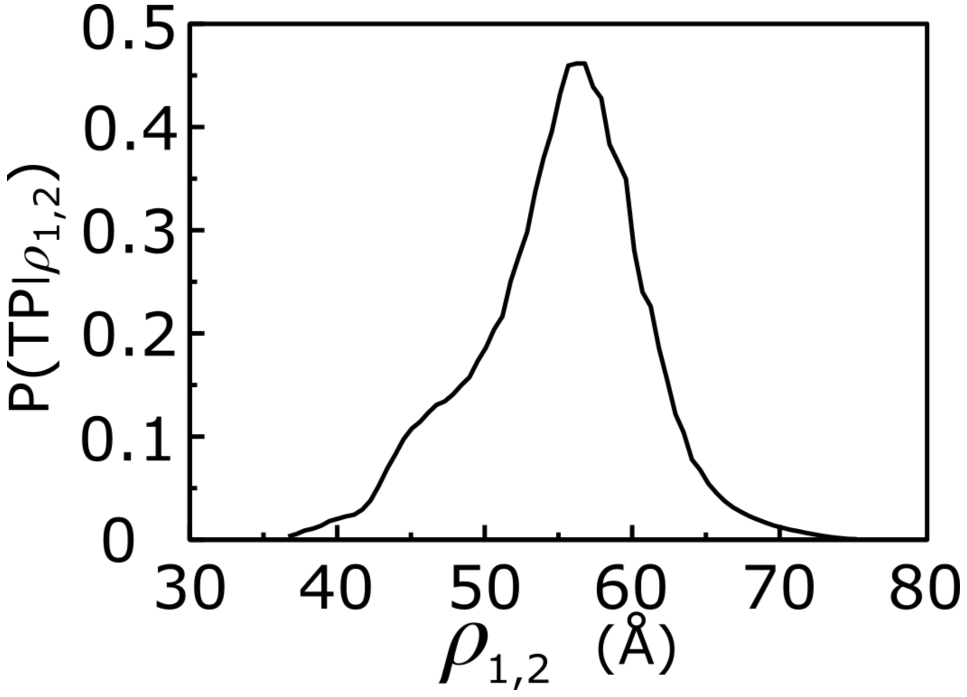

SUPPLEMENTARY FIG. 19. **The probability of being on a transition path, as a function of $\rho_{1,2}$.** To identify a suitable coordinate for describing the I1-I2 transition, transition path analysis was applied. After an extensive comparison of possible tRNA coordinates, we found that the conditional probability $P(\mathrm{TP}|\rho_{1,2})$ nearly reaches the diffusive limit of 0.5, which support the use of $\rho_{1,2}$ when calculating free-energy barriers for the I1-I2 transition.

	I1	I2	P/E	NE
I1		6.51 (74)	NC (4)	-
I2	4.66 (75)		1210 (19557)	1.49 (24)
P/E	NC (8)	551 (19557)		20 (725)
NE	-	2.14 (26)	59.5 (721)	

TABLE I. Apparent rates between states i (row) and j (column). Number of detected transitions in parentheses. "-" indicates zero events were detected. Transitions for which fewer than 20 events were observed are labeled "NC" (not calculated). Rates are given in units of $\frac{1}{10^5\tau}$, where τ is the reduced time unit. Robustness of the rates was verified by segmenting the data and recalculating the rates (Supplementary Tab. II)

	I1	I2	P/E	NE
I1		2.54	NC	-
I2	1.88		1.02	1.73
P/E	NC	1.08		1.31
NE	-	10.79	1.41	

TABLE II. The presented data set included 10 simulations of comparable duration. To verify that the calculated rates were robust to sampling, they were recalculated using 2 simulations, each (5 subsets of the data). This table shows the ratio of the maximum and minimum values obtained for each apparent rate between states i (row) and j (column). When considering forward rates, the largest ratio is only 2.54 (k_2). Since $k_3/k_2 \approx 200$, this level of uncertainty is not considered significant. While k_{-4} has a larger uncertainty, its value is not relevant when determining the rate-limiting step.

Supplementary References

[1] Jenner LB, Demeshkina N, Yusupova G, Yusupov M (2010) Structural aspects of messenger RNA reading frame maintenance by the ribosome. *Nat. Struct. Mol. Biol.* 17:555–60.

[2] Ratje A, et al. (2010) Head swivel on the ribosome facilitates translocation by means of intra-subunit tRNA hybrid sites. *Nature* 468:713–716.

[3] Whitford PC, et al. (2011) Excited states of ribosome translocation revealed through integrative molecular modeling. *Proc. Natl. Acad. Sci. USA* 108:18943–18948.

[4] Brilot AF, Korostelev AA, Ermolenko DN, Grigorieff N (2013) Structure of the ribosome with elongation factor G trapped in the pretranslocation state. *Proc. Natl. Acad. Sci. USA* 110:20994–20999.

[5] Graf M, et al. (2018) Visualization of translation termination intermediates trapped by the Apidaecin 137 peptide during RF3-mediated recycling of RF1. *Nat. Commun.* 9:1–11.

[6] Svidritskiy E, Demo G, Loveland AB, Xu C, Korostelev AA (2019) Extensive ribosome and RF2 rearrangements during translation termination. *eLife* 8:1–18.

[7] Consortium TU (2009) The universal protein resource (UniProt) in 2010. *Nucleic Acid Res.* 38:142–148.

[8] Thompson JD, Higgins DG, Gibson TJ (1994) CLUSTAL W: improving the sensitivity of progressive multiple sequence alignment through sequence weighting, position-specific gap penalties and weight matrix choice. *Nucleic Acid Res.* 22:4673–4680.

[9] Roberts E, Eargle J, Wright D, Luthey-Schulten Z (2006) MultiSeq: Unifying sequence and structure data for evolutionary analysis. *BMC. Bioinfo.* 7:1–11.

[10] Humphrey W, Dalke A, Schulten K (1996) VMD: Visual molecular dynamics. *J. Mol. Graph.* 14:33–38.

[11] Nguyen K, Whitford PC (2016) Steric interactions lead to collective tilting motion in the ribosome during mRNA-tRNA translocation. *Nat. Commun.* 7:10586–10586.

[12] Bock LV, et al. (2013) Energy barriers and driving forces in tRNA translocation through the ribosome. *Nat. Struct. Mol. Biol.* 20:1390–1396.

[13] Fischer N, Konevega AL, Wintermeyer W, Rodnina MV, Stark H (2010) Ribosome dynamics and tRNA movement by time-resolved electron cryomicroscopy. *Nature* 466:329–33.

[14] Zhou J, Lancaster L, Donohue JP, Noller HF (2019) Spontaneous ribosomal translocation of mRNA and tRNAs into a chimeric hybrid state. *Proc. Natl. Acad. Sci. USA* 116:7813–7818.

[15] Bhaskar V, et al. (2020) Dynamics of uS19 C-terminal tail during the translation elongation cycle in human ribosomes. *Cell Reports* 31:107473.

[16] Russell RB, Barton GJ (1992) Multiple protein sequence alignment from tertiary structure comparison. *Proteins* 14:309–323.

[17] Crooks GE, Hon G, Chandonia JM, Brenner SE (2004) WebLogo: A sequence logo generator. *Genome Research* 14:1188–1190.
